# Global changes in gene expression by the opportunistic pathogen *Burkholderia cenocepacia *in response to internalization by murine macrophages

**DOI:** 10.1186/1471-2164-13-63

**Published:** 2012-02-09

**Authors:** Jennifer S Tolman, Miguel A Valvano

**Affiliations:** 1 Infectious Diseases Research Group, Department of Microbiology & Immunology, University of Western Ontario, London, Ontario, N6A 5C1, Canada; 2 Department of Medicine, University of Western Ontario, London, Ontario, N6A 5C1, Canada

## Abstract

**Background:**

*Burkholderia cenocepacia *is an opportunistic pathogen causing life-threatening infections in patients with cystic fibrosis. The bacterium survives within macrophages by interfering with endocytic trafficking and delaying the maturation of the *B. cenocepacia*-containing phagosome. We hypothesize that *B. cenocepacia *undergoes changes in gene expression after internalization by macrophages, inducing genes involved in intracellular survival and host adaptation.

**Results:**

We examined gene expression by intracellular *B. cenocepacia *using selective capture of transcribed sequences (SCOTS) combined with microarray analysis. We identified 767 genes with significantly different levels of expression by intracellular bacteria, of which 330 showed increased expression and 437 showed decreased expression. Affected genes represented all aspects of cellular life including information storage and processing, cellular processes and signaling, and metabolism. In general, intracellular gene expression demonstrated a pattern of environmental sensing, bacterial response, and metabolic adaptation to the phagosomal environment. Deletion of various SCOTS-identified genes affected bacterial entry into macrophages and intracellular replication. We also show that intracellular *B. cenocepacia *is cytotoxic towards the macrophage host, and capable of spread to neighboring cells, a role dependent on SCOTS-identified genes. In particular, genes involved in bacterial motility, cobalamin biosynthesis, the type VI secretion system, and membrane modification contributed greatly to macrophage entry and subsequent intracellular behavior of *B. cenocepacia*.

**Conclusions:**

*B. cenocepacia *enters macrophages, adapts to the phagosomal environment, replicates within a modified phagosome, and exhibits cytotoxicity towards the host cells. The analysis of the transcriptomic response of intracellular *B. cenocepacia *reveals that metabolic adaptation to a new niche plays a major role in the survival of *B. cenocepacia *in macrophages. This adaptive response does not require the expression of any specific virulence-associated factor, which is consistent with the opportunistic nature of this microorganism. Further investigation into the remaining SCOTS-identified genes will provide a more complete picture of the adaptive response of *B. cenocepacia *to the host cell environment.

## Background

*Burkholderia cenocepacia *is a member of the *B. cepacia *complex (Bcc), which comprises at least 17 closely related bacterial species [1,2]. Ubiquitous in nature, Bcc bacteria thrive in the rhizosphere, fresh and marine water, and in association with amoebae, fungi, insects, plants and animals [3,4]. Members of the complex have biopesticidal and plant growth-promoting properties [5], and due to their metabolic diversity, also have the ability to degrade environmental pollutants [6]. The beneficial properties of Bcc bacteria, however, are offset by their opportunistic pathogenicity in immunocompromised individuals, especially in cystic fibrosis (CF) patients [7,8].

Bcc-infected CF patients suffer a rapid decline in lung function [9], and some develop "cepacia syndrome", a fatal condition characterized by rapid lung deterioration, acute, necrotizing pneumonia, and septicemia [8]. Resistance to almost all clinically relevant antibiotics [10] and patient-to-patient transmission further complicate treatment and prevention [11-13]. Nearly all species of the Bcc have been identified in the sputum of CF patients, but *B. cenocepacia *and *B. multivorans *are the most prevalent [14,15]. Of these, *B. cenocepacia *is associated with epidemic spread and more severe infection [15,16], and is thus the focus of this study.

Environmental strains of *B. cenocepacia *have been isolated from CF patients, indicating a single strain can occupy multiple niches [12,17]. This suggests an adaptive capacity of these bacteria to different and potentially stressful environments, which likely requires gene transcription. *B. cenocepacia *has a very large genome (~ 8 Mbp) arranged in three chromosomes and one plasmid [18]. The genome encodes many putative virulence factors, but none has been shown to be completely necessary for survival [for a recent review see reference [19]]. Therefore, little is known about the mechanisms by which *B. cenocepacia *causes disease. Bacterial survival within host cells, including epithelial cells [20] and macrophages [21-23], may potentially provide a reservoir for bacterial persistence [24]. Intracellular *B. cenocepacia *delay phagosomal maturation [21,25] and assembly of the NADPH oxidase [26,27], and alter the actin cytoskeleton [27-29]. Furthermore, intracellular *B. cenocepacia *can replicate within macrophages [30]. The bacteria-containing phagosome fuses with the lysosome and becomes acidified by 6 h post-infection [21], suggesting that *B. cenocepacia *may be able to replicate in an acidic environment.

To gain a full picture of bacterial adaption to the macrophage cell host we investigated the gene expression of intracellular bacteria upon internalization into macrophages. The study of gene expression by intracellular bacteria is challenging since bacterial mRNA, which has a short half-life, represents only a small fraction of total bacterial RNA, which itself is only a small fraction of the total RNA in infected host cells. Selective capture of transcribed sequences (SCOTS) is a technique developed to address these challenges and identify bacterial genes expressed during infection. Previous studies in other bacteria have shown that SCOTS enriches microbial transcripts without introducing a significant bias in gene expression data [31]. This technique has been used to identify genes expressed by several intracellular pathogens, including *Mycobacterium tuberculosis *[32], *Salmonella enterica *serovar Typhi [33,34], *Escherichia coli *O157:H7 [35], *Legionella pneumophila *[31], and *Ehrlichia ruminantium *[36].

In this study, we characterized gene expression by intracellular *B. cenocepacia *during an early post-infection stage in an attempt to identify gene products involved in bacterial adaptive responses to the phagosomal environment. We demonstrate that *B. cenocepacia *alters gene expression post-internalization, and that various upregulated genes are involved in bacterial entry, intracellular replication, and cytotoxicity towards the macrophage host.

## Results and discussion

### Low abundance transcripts identified by competitive enrichment

**Figure 1:**
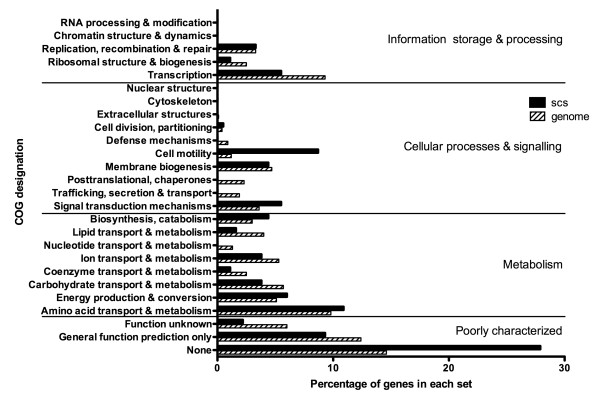
**Functional classification by COG designation of intracellular selectively captured sequences**. Profiles of the functional classes of genes are shown as a percentage of all selectively captured sequences (scs). Also shown is the functional profile of the entire *B. cenocepacia *genome.

SCOTS was used to examine gene expression by intracellular *B. cenocepacia *in murine macrophages at 4 h post-infection. This interval was selected because at 4 h post-infection most intracellular bacteria reside in phagosomes that do not fuse with the lysosome [21,26,37]; infected macrophages also display observable alterations of the actin cytoskeleton caused by the bacterial Type VI secretion system (T6SS) [27,28], which is poorly expressed in LB medium [28]. Therefore, 4 h post-infection provides a good window to document early changes in bacterial gene expression in response to the intracellular environment. As a control, we used bacteria grown for 4 h in macrophage growth medium (DMEM-10% FBS). To isolate and enrich bacterial transcripts, three rounds of SCOTS were performed on both intracellular and control bacterial cDNA samples. The resultant cDNA pools were subjected to competitive enrichment for transcripts specific to intra-macrophage bacteria. This was accomplished by pre-blocking the chromosome with both rDNA and control cDNA from non macrophage-exposed bacteria prior to capturing the cDNA from intracellular bacteria. The procedure facilitates capture of bacterial transcripts that are preferentially expressed within the phagosome. After three rounds of competitive enrichment, intracellular cDNA sequences were cloned into pUC19 and introduced into *E. coli *DH5α by transformation. DNA inserts from colonies were individually amplified by PCR and screened by Southern blot for differential hybridization to digoxigenin-labeled cDNA libraries. Many of the recombinant plasmids obtained hybridized to labeled intracellular cDNA but not to non macrophage-exposed cDNA (Additional file 1, Figure S1). One hundred eighteen unique sequences, encoding 124 proteins, were mapped to the *B. cenocepacia *genome (Additional file 2, Table S1); of these sequences, four were secondary sequences within an already identified gene, two were within a duplicated region on chromosome one, and one was a transposase gene with nine identical copies distributed throughout the genome. Identified sequences were proportionally represented in each of the three chromosomes and the plasmid: 57 sequences mapped to chromosome one, 51 to chromosome two, 14 to chromosome three, and 2 to the plasmid. Identified genes were classified into functional categories based on clusters of orthologous genes (COG) designations (Figure 1). Almost half (46%) of identified genes belonged to the category of poorly characterized genes, of which two-thirds had no COG classification. Of the remaining genes, half were involved in metabolism, one-third in cellular processes and signaling, and the remainder in information storage and processing. Several major COGs were not represented in the set, including genes encoding functions of nucleotide metabolism and transport, trafficking, secretion and transport, posttranslational modification and chaperones, and defense mechanisms. In contrast, genes classified as encoding cell motility functions were overrepresented relative to genome content.

Competitive enrichment and downstream screening do not require specialized equipment, significant financial outlay, or additional knowledge of the genome, and are thus easily and rapidly applicable to any bacterium of interest. Applying competitive enrichment to SCOTS-derived cDNA identified trends in the intracellular behavior of *B. cenocepacia*, as well as specific gene targets for further study. However, analysis of global changes in gene expression requires a high-throughput screening system.

### Global intracellular gene expression identified by microarray analysis

**Figure 2:**
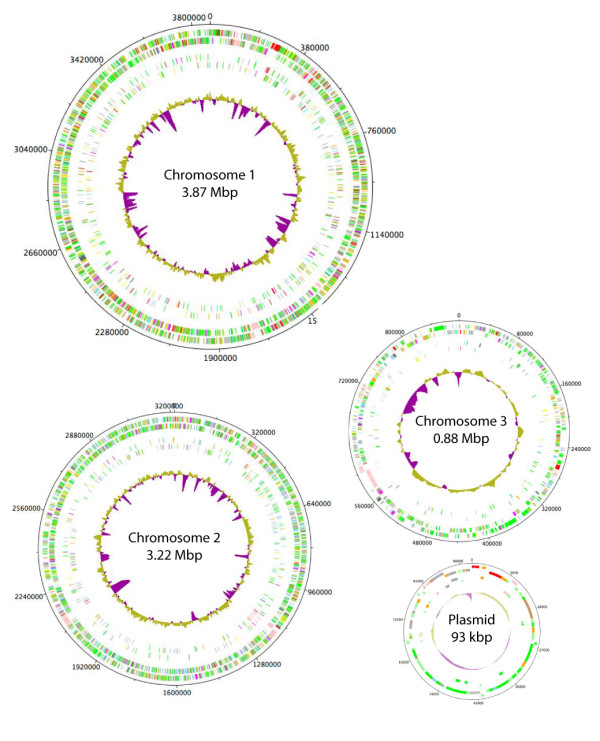
**Distribution of genes differentially expressed by intracellular *B. cenocepacia *among the four genomic replicons**. The outer pair of concentric circles represents both coding strands of the *B. cenocepacia *genome. The second pair of concentric circles represents genes with increased (outer) and decreased (inner) expression by intracellular *B. cenocepacia*. Percentage of G+C is also shown, with above average in yellow and below average in purple. Chromosome 3 is 3x scale; the plasmid is 17.5x scale.

To determine if the trends of intra-macrophage expressed genes are representative of the entire cDNA library, SCOTS-derived cDNA pools were applied to *B. cenocepacia*-specific microarrays. Each cDNA pool was hybridized against a reference sample of genomic DNA, allowing comparison between samples. Of the 118 sequences initially identified by competitive enrichment, 80 percent showed higher expression by intracellular bacteria than non-macrophage-exposed bacteria, validating the use of competitive enrichment as a preliminary investigation technique. Twenty percent of sequences identified by competitive enrichment showed significant intracellular upregulation (> 2-fold, *p *< 0.05) of the gene itself or putative transcriptionally associated gene(s). In a global comparison of gene expression between intracellular and non-macrophage-exposed bacteria, 767 genes or intergenic sequences demonstrated significant changes in expression (-2 > log2 > 2, p < 0.05) (Additional file 3, Table S2), from which 330 and 437 showed increased and decreased expression, respectively. The gene distribution among the three chromosomes and the large plasmid of *B. cenocepacia *was relatively proportionate to the size of the genetic element (Figure 2), with the exception of the plasmid, where 20% of all plasmid-encoded genes showed decreased expression by intracellular bacteria.

To validate our results we first determined how many of the genes obtained by the SCOTS method were previously identified by signature-tagged mutagenesis (STM) as essential for *in vivo *survival. In the previous study we mapped 84 unique transposon insertions in genes that were associated with attenuation of bacterial survival in a rat agar bead model of chronic lung infection [38]. Four of the STM genes were identified directly by SCOTS using competitive enrichment, and another three SCOTS-identified genes were found in genes located within a putative transcriptional unit with an STM insertion (Additional file 4, Table S3). The microarray analysis identified an additional 26 of the 84 STM genes as being more highly expressed by intracellular bacteria than non-macrophage-exposed bacteria. These results were considered significant since bacterial survival in a rat agar bead model involves more that just intra-macrophage survival.

A more direct validation of the SCOTS gene set was performed by quantitative RT-PCR using a subset of genes. Because bacterial RNA accounts for a very small proportion of the total RNA isolated from infected cells, an internal control was necessary to allow comparison across samples. Sigma factor BCAM0918, or *rpoD *(also designated *sigA *[39] or *sigE *[39,40]) was chosen due to stable expression across growth phases and conditions [41]. Despite reverse transcribing equal amounts of RNA for each sample, intracellular samples averaged 60-fold less *rpoD *transcripts than in non macrophage-exposed samples, demonstrating the scarcity of bacterial RNA relative to eukaryotic content in the intracellular sample. To account for this problem, expression of the gene of interest was first related to the internal control, and this ratio was compared between samples to give fold increase in expression in intracellular bacteria. The test genes included BCAM0314 (hypothetical protein), BCAM2141 (ABC transporter ATP-binding protein), BCAM0276 (putative universal stress protein), and BCAS0186 (putative acyl carrier protein phosphodiesterase), all of which show high expression in intracellular bacteria. BCAM1928 (putative transcription elongation factor) was chosen as an example of a gene with decreased expression in intracellular bacteria. qRT-PCR confirmed higher expression of BCAM0314, BCAM2141, BCAM0276, and BCAS0186 (Additional file 5, Figure S2); expression of negative control BCAM1928, though detectable in both samples, was far lower in intracellular bacteria than in non-macrophage-exposed bacteria, with a fold change ratio of 3.2 × 10^-3 ^± 2.8 × 10^-3^. Based on the comparison of the SCOTS results with STM data and the results of qRT-PCR, we concluded that the set of genes identified by microarray provides a good representation of the *B. cenocepacia *transcriptome in the phagosome at 4 h post infection.

**Figure 3:**
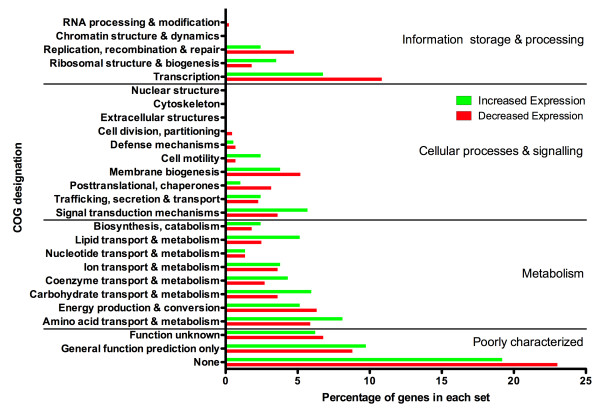
**Functional classification by COG designation of genes differentially expressed by intracellular *B. cenocepacia***. Profiles of functional classes are shown as a percentage of all genes with significantly increased (green bar) or decreased (red bar) expression (-2 > log2 > 2, *p *< 0.05).

**Figure 4:**
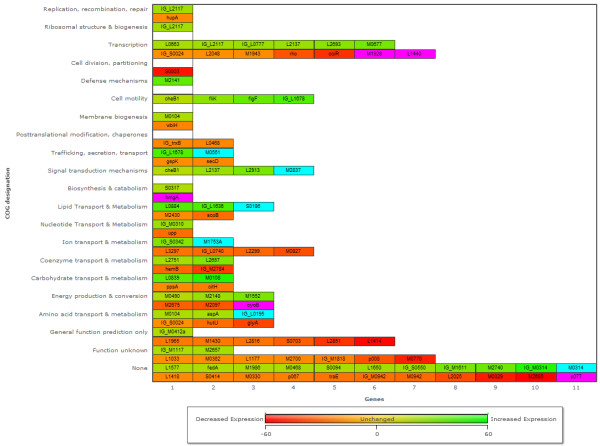
**Relative expression of genes with the greatest fold-change in intracellular *B. cenocepacia***. Heat map shows the 100 genes with the greatest ± fold-change (*p *< 0.05) in expression in intracellular bacteria relative to non-macrophage-exposed bacteria. Color scale from -60 to +60-fold change is indicated, with expression increased > 60-fold in blue, and decreased > 60-fold in purple. "BCA" has been omitted from gene names.

Differentially regulated genes identified by microarray were classified into functional categories based on COG designations (Figure 3). Globally, our results indicate that internalization of *B. cenocepacia *by macrophages causes large changes in the expression of genes associated with signal transduction mechanisms and transcription, showing that *B. cenocepacia *senses the phagosomal environment and activates mechanisms necessary for survival in a new niche. Adaptive gene expression is a continual process, as transcription-associated genes remain among the most-affected at 4 h post-infection (Figure 4). Metabolic adaptation accounts for one-third of differentially regulated genes in intracellular bacteria. In particular, genes involved in carbohydrate transport and metabolism were prominently upregulated in intracellular bacteria, accounting for 6% of all differentially upregulated genes (Figure 3). In *Leishmania*, a protozoan parasite that delays phagosomal maturation and replicates in an acidified phagolysosome, the phagolysosome contains various carbon sources and essential nutrients, but lacks carbohydrates [42], which could explain the upregulation of carbohydrate metabolism pathways. We speculate that in similar fashion, the upregulation by intracellular *B. cenocepacia *of genes involved in carbohydrate metabolism is a consequence of the low carbohydrate content in the phagosomal lumen. This also agrees with the observation that some of the most highly expressed genes in intracellular *B. cenocepacia *are those involved in amino acid transport (Figure 4). Similar findings were reported for other pathogens, which upregulate amino acid transport systems during intracellular growth [31,34,43], suggesting amino acid acquisition from the host may be a general strategy of intracellular bacteria to obtain carbon and nitrogen. However, with nearly 40% of the genes most affected by internalization falling into the poorly characterized category (Figure 4), it is likely that *B. cenocepacia *also employs novel survival strategies.

Intracellular bacteria showed decreased expression of genes involved in siderophore-mediated iron acquisition, including those encoding the ornibactin receptor, the transcriptional activator of the pyochelin receptor, and a putative pyochelin biosynthesis protein (Additional file 3, Table S2). Ornibactin contributes to colonization, bacterial persistence, and lung pathology in murine models of acute and chronic lung infection [44]. Ornibactin, however, may not be required for iron uptake by intracellular bacteria, as the phagosome appears to be rich in iron content [45-47].

The large number of genes in the *Burkholderia *genus, and specifically in *B. cenocepacia *[18], endows these bacteria with redundant nutrient uptake systems to function under different conditions and perhaps even exploit multiple forms of similar metabolites. Thus, the changes observed in the expression of metabolic genes may reflect the ability of *B. cenocepacia *to utilize resources present in the phagolysosome. Since intracellular bacteria only delay phagolysosomal fusion and reside in a phagosome that remains in contact with the endocytic pathway [21], it would be reasonable to propose that the phagosomal maturation delay allows *B. cenocepacia *to become adapted to survive in low pH environment. The results of our SCOTS analysis support the notion that intracellular *B. cenocepacia *may adapt to survive in a low pH environment. Indeed, BCAS0167, one of the two genes encoding the squalene-hopene cyclase for the synthesis of hopanoids, was strongly upregulated by intracellular bacteria. Production of cholesterol-like hopanoids in certain bacteria, including *B. cenocepacia *[48] contributes to membrane stability under acidic environments [48,49]. Furthermore, the upregulation of a putative sodium:solute symporter and a Na^+^/H^+ ^antiporter suggests that proton-driven high-affinity uptake by metabolite:proton symporters is important for intracellular *B. cenocepacia *to bring nutrients into the cell under acidic conditions [50].

### SCOTS-identified genes are involved in bacterial entry, intracellular replication, and macrophage cytotoxicity

Because survival within macrophages may contribute to the pathogenesis of *B. cenocepacia*, the role of several SCOTS-identified genes in intracellular survival was examined. Ten genes, chosen based on their high intracellular expression by microarray data or identification by competitive enrichment, were selected for deletion in the gentamicin-sensitive *B. cenocepacia *MH1K. This strain is isogenic with K56-2 but allows intracellular bacterial survival to be assessed via the gentamicin protection assay [30]. The ten deleted genes were: BCAL0124 (subunit of the flagellar regulon master regulator), BCAS0186 (putative acyl carrier protein phosphodiesterase), BCAM0434-5, (cation efflux system), BCAM2837 (response regulator component of a two-component regulatory system), BCAL1726 (putative oxidoreductase), BCAM0276 (putative universal stress protein), BCAM0411 (MgtC family protein), BCAL0340 (putative lipoprotein), BCAM1679, (putative lysylphosphatidylglycerol synthetase), and BCAM2446 (putative outer membrane porin).

**Figure 5:**
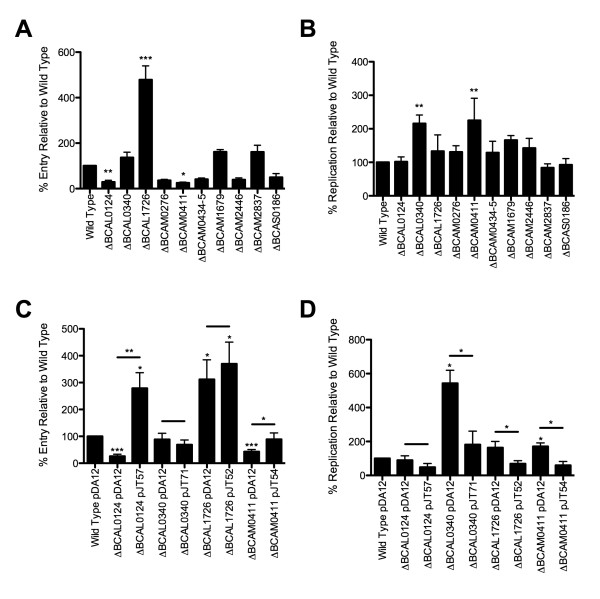
**Comparative macrophage entry and intracellular replication of parental and mutant *B. cenocepacia***. Entry (A) was calculated relative to initial inoculum, and replication (B) was calculated relative to entry; both were normalized relative to the parental control, set at 100% entry and 100% replication. Significance was determined using one-way ANOVA and Dunnett's Multiple Comparison Test. Mutants with a statistically significant difference from the parental strain (*p < 0.05, **p < 0.01, ***p < 0.001) could be complemented for both entry (C) and replication (D). Plasmid pDA12 is a vector control; pJTx is a complementation plasmid. Error bars indicate the standard error of the mean of at least three independent experiments. Vector control and complemented strains were compared using Student's t test; statistically significant differences are indicated above the lines.

The ability of mutant strains to enter and replicate within macrophages was assessed relative to the parental strain. Only BCAL0124 (*p *< 0.01) and BCAM0411 (*p *< 0.05) showed statistically significant defects in macrophage entry, while deletion of BCAL1726 resulted in a significant increase in macrophage entry (*p *< 0.001)(Figure 5A). None of the deletions negatively affected intracellular survival, but deletions in BCAL0340 and BCAM0411 increased the ability of the bacteria to replicate intracellularly (Figure 5B). With the exception of ΔBCAL1726, statistically significant differences in entry and intracellular survival could be complemented by introducing the deleted gene encoded in a plasmid under the control of a constitutive promoter (Figure 5C-D). The lack of complementation of ΔBCAL1726 suggests the possibility of a secondary mutation. Alternatively, because BCAL1726 is predicted to be the first in a four-gene operon, the deletion may affect downstream genes, thereby preventing possible complementation by BCAL1726 alone.

**Figure 6:**
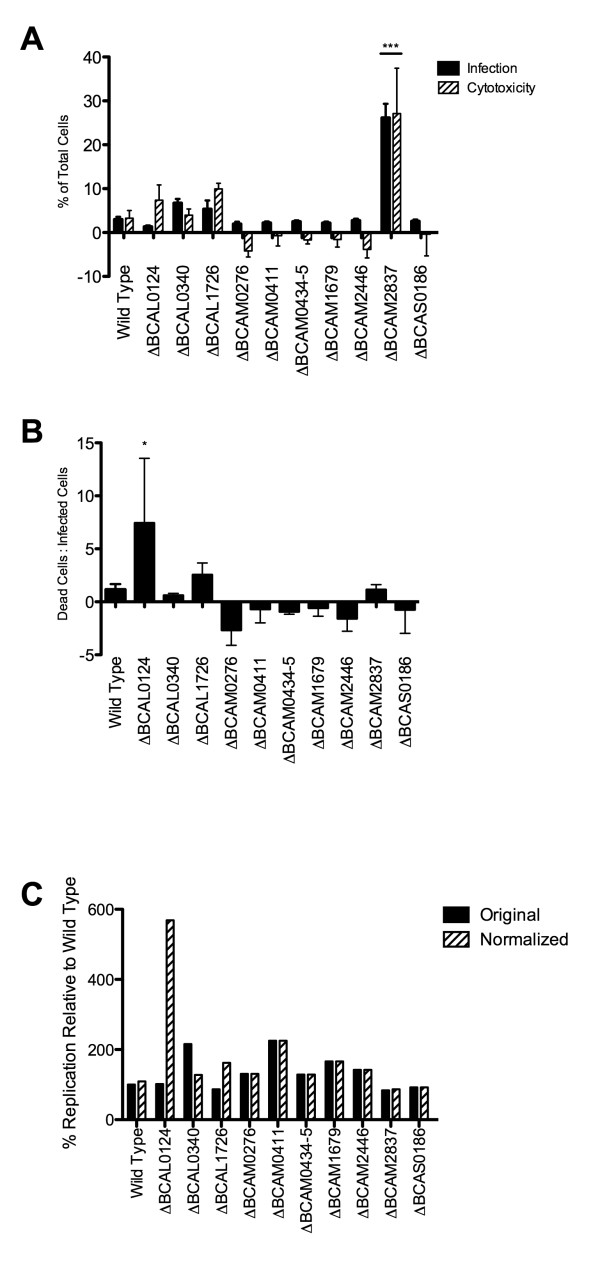
**Comparative bacterial infection, host cytotoxicity, and normalized replication of parental and mutant *B. cenocepacia***. Bacterial infection and cytotoxicity (A) are shown as a percentage of total cells. Cytotoxicity was normalized against cell death in uninfected cells. 50 000 cells were counted in each of three independent experiments. Standard error bars are indicated. Significance was determined using one-way ANOVA and Dunnett's Multiple Comparison Test. Mutants demonstrating significant difference from the wild-type are indicated (*p < 0.05, **p < 0.01, ***p < 0.001). The ratio of dead cells: infected cells (B) was used to correct intracellular replication determined by the gentamicin protection assay to give the total expected ratio of intracellular bacteria in the absence of cell death (C). Mutants with a ratio in (B) less than zero are unchanged in (C).

The results of the gentamicin protection assay may be misleading if the survival of the host cell is not considered, as bacterial cytotoxicity causing membrane damage to the host cell would allow entry of the antibiotic and result in the killing of intracellular bacteria. The viability of *Burkholderia*-infected macrophages was assayed by flow cytometry using the impermeant fluorescent dye 7-AAD, which binds DNA only in macrophages with permeabilized membranes. Macrophages were infected with GFP-expressing bacteria and assessed on three different parameters: entry, cytotoxicity, and spread. Parental *B. cenocepacia *was found in 3% of all macrophages, and was cytotoxic to macrophages at a 1:1 ratio, killing approximately 3% of all cells (Figure 6A). The only mutant displaying statistically significant differences from the parental isolate was ΔBCAM2837; despite infecting nine-fold better than the parental isolate, ΔBCAM2837 was also cytotoxic at a 1:1 ratio, killing 27% of all macrophages. Some mutants, including ΔBCAM0276 and ΔBCAM2446, appeared to prevent permeabilization of the macrophage membrane, decreasing cytotoxicity below the level seen in uninfected macrophages (Figure 6A). Of the ten mutants, only four were cytotoxic, defined as permeabilization of the host cellular membrane greater than seen in uninfected cells. The normalization of intracellular replication quantified by the gentamicin protection assay against the rate of cytotoxicity quantified by flow cytometry provided a better overall assessment of the potential role of the genes examined in our study. Normalization revealed significant intracellular replication by ΔBCAL0124 (Figure 6C). Normalization also showed that ΔBCAL0340 replicates at a rate similar to the parental isolate, but is protected from gentamicin because it is less cytotoxic. In contrast, ΔBCAL1726 is more cytotoxic than the parental strain (Figure 6C), and, despite initially infecting at a higher level, has likely been killed by gentamicin entering dying host cells. Mutants that displayed a cytotoxic ratio (Figure 6B) less than zero were not normalized, as in the absence of membrane permeability defects, intracellular bacteria would not be inhibited by gentamicin.

**Figure 7:**
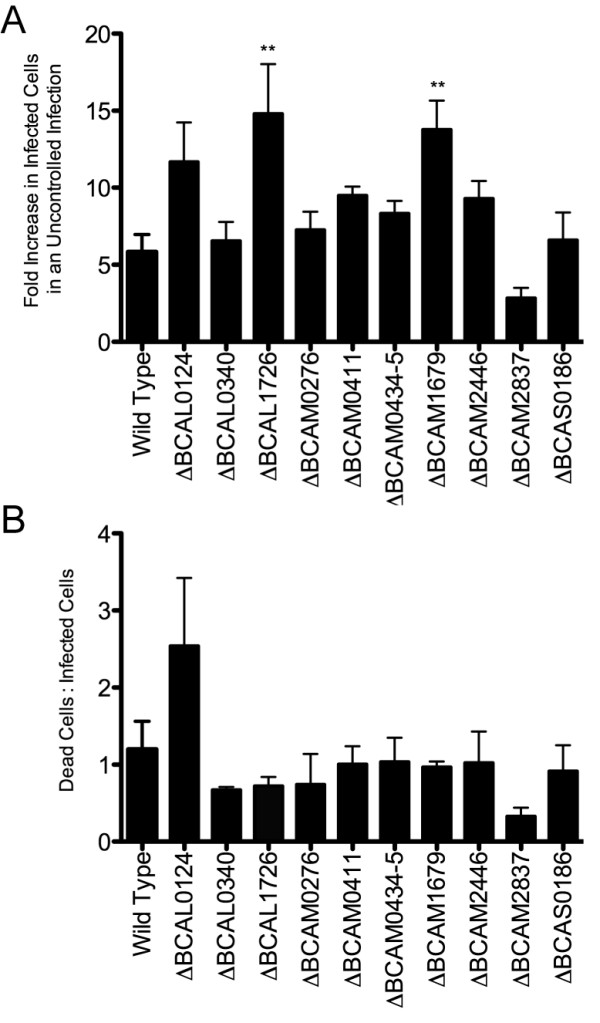
**Spread of *B. cenocepacia *and host cytotoxicity in an unchecked infection of murine macrophages**. Capacity to spread (A) is calculated as the fold-increase in infected macrophages when maintained in antibiotic-free media rather than 10 μg/ml gentamicin in the same experiment. Host cytotoxicity (B) is calculated as the ratio of total dead cells to infected cells. 50 000 cells were counted in each of three independent experiments. Standard error bars are indicated. Significance was determined using one-way ANOVA and Dunnett's Multiple Comparison Test. Mutants demonstrating significant difference from the wild-type are indicated (*p < 0.05, **p < 0.01, ***p < 0.001).

Flow cytometry also permitted investigation of possible intercellular spread of *B. cenocepacia *in an unchecked infection. After killing extracellular bacteria, infected macrophages were maintained in antibiotic-free media. Under these conditions any bacteria escaping the macrophage would be able to infect neighboring macrophages. The results of this experiment showed that the number of macrophages infected with the parental strain increased six-fold (Figure 7), suggesting that *B. cenocepacia *was able to escape the macrophage and be engulfed by neighboring macrophages. With the exception of ΔBCAM2837, the mutant strains spread at least as well as the parental isolate; both ΔBCAL1726 and ΔBCAM1679 showed significantly increased spread relative to the parental strain, more than doubling the number of infected macrophages. The ratio of dead:infected cells generally remained approximately 1:1 in an unchecked infection, suggesting that viable *B. cenocepacia *escaped from dead macrophages rather than by an active process of exocytosis. The two exceptions to this were ΔBCAL0124, which killed 2.5 cells for every cell infected at the time of analysis, and ΔBCAM2837, which killed only 30% of infected cells.

The phenotypes seen in individual gene deletions underscore several of the trends seen in the global characterization of intracellular gene expression. The importance of cellular motility suggested by the upregulation of such genes by intracellular bacteria is supported by the phenotypes seen in ΔBCAL0124 and ΔBCAM2837. BCAL0124 encodes FlhD, a subunit of the flagellar regulon master regulator necessary for expression of the flagellar structural components [51]. BCAM2837, a pseudogene in sequenced type strain J2315, is annotated as the response regulator component of a two-component regulatory system. Bioinformatic analysis shows that the protein consists of two distinct domains, one homologous to the chemotaxis protein CheC, and the second homologous to the response regulator CheY. Che proteins are typically involved in chemotaxis [52], where CheC is a phosphatase that dephosphorylates CheY [53]. The genetic organization of BCAM2837 suggests that this gene is cotranscribed with genes encoding putative diguanylate cyclase and histidine kinase proteins. *B. cenocepacia *carries three other *cheY *homologues [54,55], which are expressed in intracellular bacteria while there is one *cheC*-like gene, BCAL0136, which is not well expressed intracellularly. Therefore, we speculate that the product of BCAM2837 is part of a complex signal transduction cascade required for bacteria to move away from the phagocytic cell, which may also affect bacterial escape from dying macrophages. The mutant strain lacking BCAL0124 is non-motile, while ΔBCAM2837 is capable of swimming, but is defective in swarming (Additional file 6, Figure S3). The two mutants are significantly different in their uptake by macrophages, suggesting that the presence of flagella may be important to stimulate phagocytosis. Loss of the bacterial flagella through deletion of *fliCD *also results in a severe defect in bacterial internalization by macrophages (Additional file 7, Figure S4). The requirement of flagellin for efficient macrophage engulfment was previously noted in *B. pseudomallei *[56] and *P. aeruginosa *[57]. In contrast, bacteria with a motility defect may lose the ability to actively evade phagocytosis, resulting in increased uptake as seen in ΔBCAM2837. The two mutants also vary in their rates of intracellular replication, host cytotoxicity, and cell spread. *B. cenocepacia *lacking flagellin is unable to activate NFκB or elicit secretion of IL-8 through TLR signaling [58]. Therefore, it is possible that ΔBCAL0124 bacteria avoid activation of classical proinflammatory signaling cascades, allowing undetected intracellular replication until overwhelming the macrophage and escaping to re-infect neighboring cells. In contrast, the decreased ability to spread and lower cytotoxicity suggest that ΔBCAM2837 may kill and escape macrophages more slowly, potentially resulting from a defect in swarming motility. The importance of the flagella to the interaction between *B. cenocepacia *and macrophages is not unique, as upregulation of flagellar genes is also seen in intracellular *L. pneumophila *[31].

Genes involved in membrane biogenesis and modification, as well as trafficking across the membrane were highly regulated by intracellular bacteria (Figure 3). General systems involved in the biogenesis of membrane proteins such as the first gene of the Tat system, several genes in the Sec system, and the signal recognition particle RNA showed decreased expression by intracellular bacteria. In contrast, increased expression of T6SS genes was seen in intracellular bacteria. Deletion of BCAL0340, encoding a putative lipoprotein central to the T6SS cluster, promotes replication of intracellular bacteria (Figure 5) but does not inactivate the T6SS, as evidenced by the appearance of actin-rich protrusions in infected macrophages, a typical phenotype of a functionally active T6SS. In a previous study, three transposon mutants within the T6SS cluster showed attenuated survival in an *in vivo *rat model of chronic lung infection [38], corroborating the importance of a functional T6SS to infection. Genes involved in modification of the membrane were also highly expressed by intracellular bacteria. BCAM1679 encodes a putative lysylphosphatidylglycerol (Lys-PG) synthetase which adds lysine to PG, thereby lowering the net negative charge of the cellular envelope, already lowered in *B. cenocepacia *by the constitutive presence of 4-amino-4-*deoxy*-L-arabinose linked to lipid A [59]. Lys-PG may also decrease permeability to cations and protons by raising the surface potential of the membrane, increase membrane fluidity, inhibit genomic DNA replication, and decrease activity of the host defense factor phospholipase A2 (PLA2) (reviewed in [60]). Deletion of BCAM1679 caused extensive spread in an unchecked infection (Figure 7). Since secreted PLA2 can hydrolyze the membrane of cells undergoing early stages of apoptosis [61], it is possible that macrophages infected with ΔBCAM1679 undergo apoptosis and intracellular bacteria could escape from apoptotic cells to re-infect neighboring cells.

Previous studies have demonstrated that *B. cenocepacia *is cytotoxic to epithelial cells [62-64] and causes necrosis of dendritic cells [65]. Our results indicate that the same is true for macrophages. The mechanism of macrophage cell death induction by *B. cenocepacia *is under investigation in our laboratory [66,67]. Pilin-mediated apoptosis induced by *B. cenocepacia *in infected epithelial cells has been documented and shown to depend on the bacterial load [62]. However, production of IL-1β by infected macrophages indicates inflammasome-mediated caspase-1 activation, which can lead to pyroptosis [67]. Pyroptosis also induces lysosome exocytosis, whereby any bacteria residing within a lysosome are freely delivered to the extracellular milieu as the lysosome fuses with the macrophage plasma membrane, which would explain the mechanism of bacterial release and reinfection of neighboring macrophages.

## Conclusions

Our results indicate that the intracellular survival of *B. cenocepacia *in macrophages is associated with profound changes in metabolic activity and motility rather than the expression of more traditional "virulence-specific" genes. These observations are consistent with the ability of *B. cenocepacia *to adapt and survive in a broad range of niches. Our data also show that the delay of phagosomal maturation provides sufficient time for intracellular bacteria to undergo changes in gene expression that likely provide a growth advantage in the phagolysosomal compartment. Therefore, *B. cenocepacia *is an opportunistic pathogen with the capacity to adapt to survive intracellularly until bacteria can escape back to the extracellular milieu.

## Methods

### Bacterial strains, plasmids, media, and growth conditions

Bacterial strains and plasmids used in this study are listed in Table 1. Bacteria grew at 37°C in Luria-Bertani (LB; Difco) broth with agitation or on LB plates with 1.6% Bacto agar. *Escherichia coli *cultures were supplemented as required with 20 μg/ml tetracycline, 50 μg/ml trimethoprim, and 40 μg/ml kanamycin; *B. cenocepacia *cultures were supplemented as required with 100 μg/ml tetracycline and 100 μg/ml trimethoprim. Plasmids were conjugated into *B. cenocepacia *by triparental mating at 37°C using *E. coli *DH5α carrying the helper plasmid pRK2013 [68]. *E. coli *donor and helper strains were selected against with gentamicin (50 μg/ml) or ampicillin (100 μg/ml) and polymyxin B (25 μg/ml).

**Table 1:** Strains, plasmids, and cell lines used in this study

Strain, cell line, or plasmid	Relevant characteristics^a^	Source or reference
*Escherichia coli*		
DH5α	F^-^, ɸ 80 *lacZ *DM15 Δ(*lacZYA-argF*)*U169 endA1 recA1 hsdR17 *(r_K_^- ^m_K_^+^) *supE44 thi-1 *Δ*gyrA96 relA1*	Laboratory stock
SY327	*araD *Δ(*lac pro*) *argE*(*Am*) *recA56 rifr nalA*, λ *pir*	[75]
*Burkholderia cenocepacia*		
J2315	Epidemic strain ET12 clone, CF clinical isolate	P.A. Sokol
K56-2	Epidemic strain ET12 clone, CF clinical isolate	BCRRC^b^
MH1K	K56-2 ΔBCAL1674-6	[30]
JST19	MH1K ΔBCAL0124	This study
JST71	MH1K ΔBCAS0186	This study
JST75	MH1K ΔBCAM2837	This study
JST128	MH1K ΔBCAL1726	This study
JST130	MH1K ΔBCAM0276	This study
JST132	MH1K ΔBCAM0411	This study
JST134	MH1K ΔBCAM2446	This study
JST136	MH1K ΔBCAM0434-5	This study
JST190	MH1K ΔBCAL0340	This study
JST194	MH1K ΔBCAM1679	This study
Macrophage cell lines		
ANA-1	C57BL/6 murine bone marrow-derived macrophages	[76]
RAW 264.7	BALB/c murine macrophage cell line	ATCC^c^
Plasmid		
pDA12	*ori_pBBR1_*, Tet^R^, *mob^+^, P_dhfr_*	[28]
pDA42	pDA12, *eGFP*	[28]
pDAI-SceI-SacBN	*ori_pBBR1_*, Tet^R^, *mob^+^, P_dhfr_, sacB*, encodes I-SceI endonuclease	[30]
pDelbcsM	pGPI-SceI with regions flanking BCAL0340	[77]
pGPI-SceI	*ori_R6K_*, Tp^R^, *mob^+^*, carries I-SceI cut site	[74]
pJT25	pUC19 with K56-2 rDNA (23S, 16S, 5S), Ap^R^	This study
pJT31	pGPI-SceI with regions flanking BCAL0124	This study
pJT41	pGPI-SceI with regions flanking BCAS0186	This study
pJT42	pGPI-SceI with regions flanking BCAM2837	This study
pJT45	pGPI-SceI with regions flanking BCAL1726	This study
pJT46	pGPI-SceI with regions flanking BCAM0276	This study
pJT47	pGPI-SceI with regions flanking BCAM0411	This study
pJT48	pGPI-SceI with regions flanking BCAM2446	This study
pJT49	pGPI-SceI with regions flanking BCAM0434-5	This study
pJT52	pDA12, BCAL1726	This study
pJT54	pDA12, BCAM0411	This study
pJT57	pDA12, BCAL0124	This study
pJT71	pDA12, BCAL0340	This study
pJT72	pGPI-SceI with regions flanking BCAM1679	This study
pRK201	RK2 derivative, Km^R^, *mob*^+^, *tra*^+^, *ori*_colE1_	[68]
pUC19	*ori_pMB1_*, Ap^R^, *mob^+^, P_lac _*	[78]

^a ^Tp, trimethoprim; Tet, tetracycline; Ap, ampicillin; Km, kanamycin

^b ^*B. cepacia *complex Research and Referral Repository for Canadian CF Clinics

^c ^American Type Culture Collection

### Molecular techniques

DNA manipulations were performed as in [69]. DNA was amplified by PCR in a PTC-221 DNA engine (MJ Research) with *Taq *DNA polymerase or HotStar DNA polymerase (Qiagen). Amplification of *B. cenocepacia *DNA was aided by including the Qiagen Q solution. DNA sequencing was performed at the York University Core Molecular Biology and DNA Sequencing Facility in Toronto, Ontario, Canada. Restriction enzymes, T4 DNA ligase (Roche Diagnostics) and Antarctic alkaline phosphatase (New England Biolabs) were used as recommended by manufacturers. Transformation of *E. coli *SY327 and DH5α was done by the calcium chloride protocol [70].

### Cell culture and infection

Macrophage cell lines (Table 1) were maintained in Dulbecco's modified Eagle medium (DMEM; Wisent) supplemented with 10% fetal bovine serum (FBS; Wisent) at 37°C in a 95% humidified atmosphere with 5% CO_2_. For macrophage infection, cells were seeded in a 12-well plate at 1.25 × 10^5 ^cells per well or a 6-well plate at 2.5 × 10^5 ^cells per well and grown 15 h. Bacterial cultures grown at 37°C for 16 h were washed twice and resuspended in DMEM-10% FBS. Macrophage monolayers were washed with phosphate-buffered saline (PBS; Wisent). Bacteria were added at a multiplicity of infection (MOI) of 50:1 or 10:1 in 1-2 ml DMEM-10% FBS. Plates were centrifuged 1 min at 300 × *g *and incubated at 37°C, 95% humidity and 5% CO_2_.

### RNA extraction, cDNA synthesis, and amplification

RNA was extracted from two 6-well plates containing equal numbers of *B. cenocepacia*, differing only in the presence (intracellular (I) RNA) or absence (non-macrophage-exposed RNA) of macrophages. At 4 h post-infection, macrophage monolayers were washed three times with PBS and lysed in 1 ml cold deionized H_2_O. Non macrophage-exposed bacteria were resuspended and collected. For each condition, cells from 6 wells were resuspended in 1 mg/ml lysozyme (Roche). Total RNA was extracted using TRIzol (Invitrogen) and treated with RNase-free DNase (Roche) according to the manufacturer's instructions. RNA purity, integrity, and concentration were determined by PCR, agarose gel electrophoresis, and A_260_/A_280 _spectrophotometer readings, respectively. A 5 μg RNA sample from each condition ("intracellular" and "non macrophage-exposed") was converted to first-strand cDNA by random priming with Transcriptor reverse transcriptase (Roche) according to the manufacturer's specifications. Primers had a defined 5' terminal sequence and a 3' random nonamer; different terminal sequences were used for intracellular (I-3025) and non macrophage-exposed RNA (N-3017) (Additional file 8, Table S4). cDNAs were double-stranded using Klenow fragment (Roche) as described in [71]; cDNA libraries were amplified for 25 cycles using defined primers I-3032 (intracellular) or N-3033 (non macrophage-exposed).

### Selective capture of transcribed sequences

The SCOTS protocol was carried out as in [72]. Briefly, 0.3 μg of denatured, sonicated, biotinylated *B. cenocepacia *K56-2 chromosome were mixed with 5 μg denatured ribosomal DNA fragments (sonicated pJT25; Table 1) and hybridized 30 min at 68°C to pre-block rRNA-encoding DNA regions. 3 μg of cDNA were denatured and re-annealed 30 min at 68°C to remove abundant transcripts. cDNA and chromosomal DNA were combined and hybridized 24 h at 68°C. Chromosome-cDNA hybrids were removed from solution with streptavidin-coated magnetic beads (Invitrogen). Captured cDNA was eluted, precipitated, and amplified using library-specific defined primers (Additional file 8, Table S4). For each condition, 10 parallel first-round reactions were done to maximize the cDNA sample diversity; the resultant cDNA was pooled for each condition and two subsequent rounds of SCOTS carried out. The final cDNA libraries were used both for competitive enrichment hybridization and as probes for *B. cenocepacia *microarrays.

### Competitive enrichment

To preferentially enrich for intramacrophage-expressed transcripts, 0.3 μg of *B. cenocepacia *K56-2 chromosome was pre-blocked with both 5-μg rDNA and 10 μg of denatured triple-SCOTS enriched non macrophage-exposed cDNA (above). 3 μg of triple-SCOTS enriched intracellular cDNA (above) was denatured and re-annealed 30 min at 68°C to remove abundant transcripts. cDNA and blocked chromosomal DNA were combined and hybridized 18 h at 68°C. Chromosome-cDNA hybrids were removed from solution with streptavidin-coated magnetic beads. Captured cDNA was eluted, precipitated, and amplified using intracellular library-specific defined primer I-3032. Following three rounds of enrichment, cDNA were digested with restriction enzyme *Xba*I, cloned into *Xba*I-digested, dephosphorylated pUC19, and transformed into *E. coli *DH5α. Individual cDNA inserts were screened by Southern blot for hybridization to DIG-labeled cDNA libraries. cDNA that hybridized to intracellular cDNA, but not to non macrophage-exposed cDNA, were sequenced and identified by BLAST analysis.

### Microarray experimental design and analysis

SCOTS cDNA or *B. cenocepacia *J2315 genomic DNA was labeled with CyScribe™ Array CGH Labeling Kit (GE Healthcare) according to the manufacturer's protocols. SCOTS cDNA was labeled with Cy5 and genomic DNA with Cy3; cDNA samples were mixed, hybridized to custom *B. cenocepacia *microarrays (Agilent) according to the Agilent 60-mer oligonucleotide microarray processing protocol, and scanned. Labeling of cDNA, hybridization, and scanning of arrays were performed by the Mahenthiralingam Laboratory, Cardiff University, Wales. Microarray data analysis was done using GeneSpring GX 7.3.1 (Agilent). Raw data was preprocessed via the enhanced Agilent FE import prior to per-spot and per-chip normalizations for each array. Feature intensity varied across the six arrays. Statistical analysis between intracellular and non-macrophage-exposed conditions was performed using a paired Student's *t*-test; genes were considered differentially expressed at an intracellular:non macrophage-exposed ratio of ± 2-fold with a *p *< 0.05.

The microarray dataset has been deposited in the ArrayExpress database http://www.ebi.ac.uk/arrayexpress/ under accession number E-MEXP-3408.

### Quantitative reverse transcriptase PCR (qRT-PCR)

To validate microarray data, five genes with altered expression (4 upregulated: BCAM0314, BCAM2141, BCAM0276, and BCAS0186; and 1 downregulated: BCAM1928) were examined individually using qPCR. Sigma factor gene *rpoD *(BCAM0918) was used as a reference gene. Oligonucleotide primers for each gene (Additional file 8, Table S4) were designed with Primer3 [73]. Total RNA was isolated as described above. cDNA was synthesized using random hexamers (Invitrogen) and Transcriptor reverse transcriptase. Quantitation and melting curve analyses for qPCR were performed using the Rotor Gene 6000 (Corbett Life Science) with FastStart SYBR Green (Roche) according to the manufacturer's instructions. Expression of the target gene was normalized to the reference gene for each condition, allowing inter-condition comparison. Data shown are representative of at least two independent experiments.

### Mutagenesis of *B. cenocepacia *K56-2

The I-*Sce*I homing endonuclease system was used to construct unmarked deletions of genes or gene clusters in *B. cenocepacia *K56-2 [74]. Briefly, regions flanking the gene or genes to be deleted were amplified with gene-specific primers containing restriction sites (Additional file 8, Table S4). Upstream amplicons were digested with restriction enzymes *Xba*I-*Cla*I; downstream amplicons were digested with *Cla*I-*Eco*RI. Both upstream and downstream amplicons were cloned into *Xba*I-*Eco*RI digested, de-phosphorylated pGPI-*Sce*I, giving rise to mutagenesis plasmids (Table 1). Mutagenesis plasmids were introduced into either *B. cenocepacia *K56-2 or MH1K by triparental mating. Plasmid pDAI-*Sce*I-SacBN, encoding the homing endonuclease, was conjugated into single crossover mutants, causing a double-strand break resolved by either a second crossover, yielding mutant genotype, or by reversion to wild-type. Exconjugants were screened by PCR, and confirmed mutants were plated on LB with 5% sucrose to cure pDAI-*Sce*I-SacBN. PCR confirmed that the deletion had occurred, yielding gentamicin-sensitive mutants (Table 1).

### Complementation experiments

To complement single gene deletions, wild-type genes were PCR amplified from *B. cenocepacia *K56-2 with gene-specific primers (Additional file 8, Table S4) and the following thermal cycling conditions: 95°C for 5 minutes, 30 cycles of 95°C for 45 s, 60°C for 45 s, and 72°C for 90 s, and a final extension at 72°C for 10 min. The resulting amplicon was digested with restriction enzymes *Xba*I and *Nde*I, and ligated into *Xba*I/*Nde*I-digested, dephosphorylated pDA12, giving rise to a complementation plasmid (Table 1), which could be introduced to the mutant strain through conjugation. pDA12 was used as a vector control in all experiments. Statistically significant differences between vector control and complemented strains were determined using Student's t test.

### Gentamicin protection assays

Thirty min post-infection, *B. cenocepacia*-infected cells were washed three times with PBS and DMEM-50 μg/ml gentamicin was added to kill extracellular bacteria. After 30 min, the media was replaced with DMEM-10 μg/ml gentamicin. Infected monolayers were washed with PBS, and lysed with 0.1% Triton X-100 (Sigma) in PBS at 1 and 24 h post-infection. Surviving bacteria were enumerated by bacterial plate count (CFU). Bacterial entry was calculated as a percentage of initial inoculum. Intracellular replication was calculated as a percentage of bacterial entry for each strain. To compare between experiments, percentage recovery was normalized against the parental control, set as 100% entry and replication. Statistical analysis was performed using one-way ANOVA and Dunnett's Multiple Comparison Test.

### Flow cytometry

*B. cenocepacia *expressing eGFP from the plasmid pDA42 [28] were infected as above in 12-well plates at a multiplicity of 50:1. At 2 h post-infection cells were washed three times with PBS and DMEM-50 μg/ml gentamicin was added to kill extracellular bacteria. After 30 min, the medium was replaced with DMEM with and without gentamicin (10 μg/ml). Infected monolayers were washed with PBS, and removed with cold 0.04% EDTA in PBS at 24 h post-infection. Cells maintained in media with gentamicin represented initial infection, while cells maintained in antibiotic-free media represented uncontrolled infection. Macrophages were collected by centrifugation for 5 min at 400 × *g *and 4°C. Cells were resuspended in cold PBS with 2.5 μg/ml 7-aminoactinomycin D (7AAD; Invitrogen) and maintained on ice. Samples were enumerated using a FACSCalibur with CellQuest Pro acquisition software (Becton Dickinson). Data analysis was done with FlowJo (Tree Star, Inc.). All results were normalized against uninfected controls. Statistical analysis was performed using one-way ANOVA and Dunnett's Multiple Comparison Test.

## Authors' contributions

JST conceived the project, designed and conducted the experiments, analyzed data, wrote the manuscript, and edited the manuscript. MAV conceived the project, obtained funding for the project, designed the experiments, analyzed data, and edited the manuscript. All authors read and approved the final manuscript.

## Supplementary Material

Additional file 1 **Figure S1-Bacterial transcripts specific to intracellular *B. cenocepacia *can be identified by differential hybridization**. Click here for file

Additional file 2 **Table S1-Intracellular-specific selectively captured sequences**. Click here for file

Additional file 3 **Table S2-Genes with significantly higher or lower expression by intracellular *B. cenocepacia***. Genes included show greater than 2-fold change in expression (*p *< 0.05) in intracellular bacteria relative to non-macrophage-exposed bacteria. The first sheet contains genes with increased intracellular expression, the second genes with decreased intracellular expression. Click here for file

Additional file 4 **Table S3-*In vivo *essential genes with increased expression in intracellular bacteria**. Click here for file

Additional file 5 **Figure S2-Fold change gene expression of selected SCOTS-identified genes measured by qRT-PCR**. Click here for file

Additional file 6 **Figure S3-ΔBCAL0124 and ΔBCAM2837 are defective in motility**. Click here for file

Additional file 7 **Figure S4-Flagellin is necessary for efficient bacterial entry into macrophages**. Click here for file

Additional file 8 **Table S4-Oligonucleotide primers used in this study**. Click here for file
