# Supplementary material for: Global changes in gene expression by the opportunistic pathogen Burkholderia cenocepacia in response to internalization by murine macrophages
Source: BMC Genomics. 2012 Feb 9;13:63. doi: 10.1186/1471-2164-13-63 (PMC3296584; doi:10.1186/1471-2164-13-63)
Supplement: Additional file 1 — Figure S1-Bacterial transcripts specific to intracellular B. cenocepacia can be identified by differential hybridization. [file 1471-2164-13-63-S1.DOC]

**
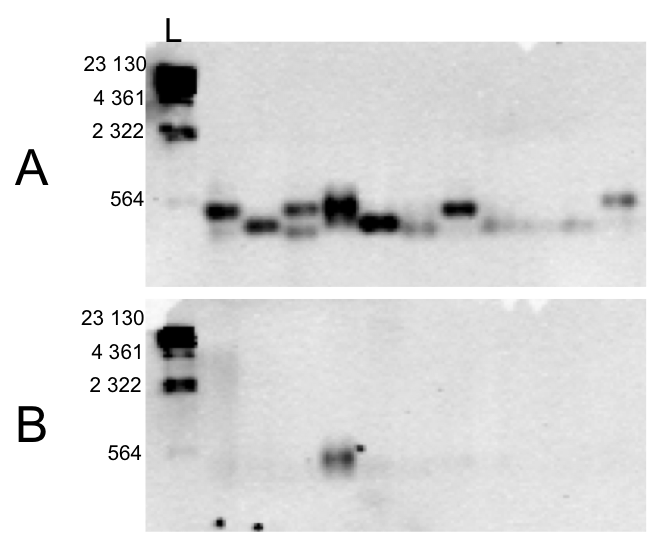
**

**Figure S1 – Bacterial transcripts specific to intracellular *B. cenocepacia* can be identified by differential hybridization.** Selectively captured sequences isolated from intracellular bacteria were cloned, and cDNA inserts were PCR amplified for each recombinant plasmid. Inserts were hybridized with DIG-labelled SCOTS-derived *B. cenocepacia* cDNA pools from (A) intracellular bacteria or (B) non-macrophage-exposed bacteria. Each lane represents the cDNA insert amplified from a single recombinant plasmid. L indicates the DIG-labelled molecular weight marker, with values in bp.
