# Supplementary material for: Global changes in gene expression by the opportunistic pathogen Burkholderia cenocepacia in response to internalization by murine macrophages
Source: BMC Genomics. 2012 Feb 9;13:63. doi: 10.1186/1471-2164-13-63 (PMC3296584; doi:10.1186/1471-2164-13-63)
Supplement: Additional file 2 — Table S1-Intracellular-specific selectively captured sequences. [file 1471-2164-13-63-S2.DOC]

**Table S1: Intracellular-specific selectively captured sequences**

|  |  | **COG** | **Gene^a^** | **Function** |
| --- | --- | --- | --- | --- |
| Metabolism | Amino Acid Transport & Metabolism | E | BCAL0017 | putative branched-chain amino acid ABC transporter periplasmic protein |
|  |  | E | ***BCAL1822*** | putrescine transport system permease protein |
|  |  | E | BCAL2224 | glutamine synthetase |
|  |  | E | BCAL3263 | conserved hypothetical protein |
|  |  | E | BCAM0187 | putative 2-isopropylmalate synthase |
|  |  | E | BCAM1172 | gamma-glutamylputrescine oxidoreductase |
|  |  | E | BCAM2237 | putative 2,2,-dialkylglycine decarboxylase |
|  |  | E | BCAM2276 | putative FAD dependent oxidoredutase |
|  |  | E | BCAM2502 | 3-dehydroquinate dehydratase |
|  |  | E - | BCAM2486 BCAM2487 | putative membrane protein putative membrane protein |
|  |  | GEPR | BCAM0068 | Major Facilitator Superfamily protein |
|  |  | GEPR | BCAM1460 | Major Facilitator Superfamily protein |
|  | Energy Production & Conversion | C C | BCAL0850 BCAL0851 | glycolate permease putative iron-sulphur cluster containing protein |
|  |  | C | BCAL0032 | ATP synthase B chain |
|  |  | C | BCAL0205 | NAP-dependent malic enzyme |
|  |  | C | BCAL2208 | dihydrolipoamide acetyltransferase component of pyruvate dehydrogenase complex |
|  |  | C | BCAL2339 | NADH dehydrogenase I chain F |
|  |  | S C | BCAS0199 BCAS0201 | putative transporter protein-Dct family putative FAD dependent oxidoreductase |
|  | Carbohydrate Transport & Metabolism | G | BCAL1010 | beta-hexosaminidase 2 |
|  |  | G | BCAL2860 | beta-hexosaminidase 1 |
|  |  | G | BCAL3240 | putative capsular polysaccharide transporter ATP-binding protein |
|  |  | G | ***BCAM1245*** | putative phosphoenolpyruvate phosphomutase/sugar nucleotidyltransferase |
|  |  | G | BCAM2586 | putative lactonase |
|  |  | GEPR | BCAM0068 | Major Facilitator Superfamily protein |
|  |  | GEPR | BCAM1460 | Major Facilitator Superfamily protein |
|  | Coenzyme Transport & Metabolism | H | ***BCAL3054*** | 6,7-dimethyl-8-ribityllumazine synthase |
|  | Ion Transport & Metabolism | P | BCAL3023 | putative chloride-channel protein |
|  |  | P | BCAM0435 | cation efflux system protein |
|  |  | P - | BCAS0249 BCAS0250 | chromate resistance transport protein conserved hypothetical protein |
|  |  | GEPR | BCAM0068 | Major Facilitator Superfamily protein |
|  |  | GEPR | BCAM1460 | Major Facilitator Superfamily protein |
|  | Lipid Transport & Metabolism | I | BCAM0151 | conserved hypothetical protein (fragment) |
|  |  | I | BCAM2433 IG | upstream region from putative acyl-CoA dehydrogenase |
|  |  | I | **BCAS0186** | putative acyl carrier protein phosphodiesterase |
|  |  | IQ | BCAL3474 | long-chain-fatty-acid—CoA ligase |
|  | Biosynthesis & Catabolism | Q | BCAL1647 | putative oligosaccharide ABC transporter ATP-binding protein |
|  |  | Q | BCAS0371 | conserved hypothetical protein |
|  |  | Q Q | BCAM2123 BCAM2124 | putative 4-oxalocrotonate decarboxylase 2-keto-4-pentenoate hydratase |
|  |  | IQ | BCAL3474 | long-chain-fatty-acid—CoA ligase |
| Cellular Processes & Signalling | Cell Division & Partitioning | D | ***BCAL3458*** | cell division protein FtsA |
|  | Signal Transduction Mechanisms | T | ***BCAL1536*** | putative sigma-54 related transcriptional regulatory protein |
|  |  | T | BCAL1975 | putative diguanylate cyclase |
|  |  | T | BCAM1160 | putative cyclic-di-GMP signaling protein |
|  |  | T | pBCA055 | putative membrane protein |
|  |  | T - | BCAL1599b BCAL1601 | hypothetical phage protein (BcenGI7) hypothetical phage protein (BcenGI7) |
|  |  | NT | BCAM2564 | putative aerotaxis receptor |
|  |  | NT NT | BCAL0133 **BCAL0134** | putative chemoreceptor glutamine deamidase cheD chemotaxis response regulator protein-glutamate methylesterase 1 |
|  | Membrane Biogenesis | M | BCAL0110 | putative aminotransferase |
|  |  | M | BCAL3239 | glucosyltransferase |
|  |  | M | BCAL3244 | glycosyltransferase |
|  |  | M | BCAM2253 | RHS-family protein |
|  | Cell Motility | N | ***BCAL0571*** | flagellar P-ring protein precursor |
|  |  | N | BCAM1357 | putative gluconate 2-dehydrogenase flavoprotein subunit |
|  |  | N | BCAM1572 IG | upstream region from methyl-accepting chemotaxis protein |
|  |  | N | **BCAS0104 IG** | upstream region from A-type flagellar hook-associated protein 2 |
|  |  | N | ***BCAL0566*** | basal-body rod modification protein FlgD |
|  |  | N | **BCAL0576** | flagellar hook-associated protein 1 |
|  |  | N | BCAM0779 | putative methyl-accepting chemotaxis protein |
|  |  | N | BCAM2563 | methyl-accepting chemotaxis protein |
|  |  | NT | BCAM2564 | putative aerotaxis receptor |
|  |  | NT NT | BCAL0133 **BCAL0134** | putative chemoreceptor glutamine deamidase cheD chemotaxis response regulator protein-glutamate methylesterase 1 |
| Information Storage & Processing | Ribosomal Structure & Biogenesis | J R | BCAL0983 BCAL0984 | ribosomal large subunit pseudouridine synthase C 2 haloacid dehalogenase-like hydrolase |
|  |  | R J | BCAL2886 BCAL2887 | haloacid dehalogenase-like hydrolase ribosomal large subunit pseudouridine synthase C 2 |
|  | Transcription | K | BCAL1182 | TetR family regulatory protein |
|  |  | K | BCAL3478 | putative RNA polymerase sigma factor |
|  |  | K | **BCAM1810** | putative cold shock protein |
|  |  | K | BCAS0258 | GntR family regulatory protein |
|  |  | K - | BCAL1403 BCAL1403A | LysR family regulatory protein putative membrane protein |
|  |  | K R | BCAM1848 BCAM1849 | IclR family regulatory protein putative hydrolase |
|  | Replication, Recombination, Repair | L | BCAL0420 | type I restriction component of type I restriction-modification system |
|  |  | L | BCAL0770 | error-prone DNA polymerase |
|  |  | L | BCAL3371 | putative DNA polymerase III delta subunit |
|  |  | L | BCAM0780 | putative helicase |
|  |  | L | BCAM0992 | putative DNA methylase |

| Poorly Characterized | General Function Prediction Only | R | BCAL0772 | AraC family regulatory protein |
| --- | --- | --- | --- | --- |
|  |  | R | BCAL2978 | NAD-dependent formate dehydrogenase alpha subunit |
|  |  | R | **BCAM0949** | exported lipase LipA |
|  |  | R | BCAS0252 | DJ-1/PfpI family protein |
|  |  | R R | BCAL1643 BCAL1644 | putative ATP/GTP binding protein permease protein |
|  |  | R J | BCAL2886 BCAL2887 | haloacid dehalogenase-like hydrolase ribosomal large subunit pseudouridine synthase C 2 |
|  |  | R S | ***BCAL0343* *BCAL0344*** | putative T6SS protein TssD putative T6SS protein TssE |
|  |  | J R | BCAL0983 BCAL0984 | ribosomal large subunit pseudouridine synthase C 2 haloacid dehalogenase-like hydrolase |
|  |  | K R | BCAM1848 BCAM1849 | IclR family regulatory protein putative hydrolase |
|  |  | - R | BCAM1569 BCAM1570 | putative BNR/Asp-box protein alcohol dehydrogenase |
|  |  | GEPR | BCAM0068 | Major Facilitator Superfamily protein |
|  |  | GEPR | BCAM1460 | Major Facilitator Superfamily protein |
|  | Function Unknown | S | ***BCAL0351*** | putative T6SS protein TssM |
|  |  | S | BCAL1165 | conserved hypothetical protein |
|  |  | S | BCAL1664 | conserved hypothetical protein |
|  |  | S | BCAM2254 IG | upstream region from putative exported protein |
|  |  | S | BCAS0667 | conserved hypothetical protein |
|  |  | S C | BCAS0199 BCAS0201 | putative transporter protein-Dct family putative FAD dependent oxidoreductase |
|  |  | R S | ***BCAL0343* *BCAL0344*** | putative T6SS protein TssD putative T6SS protein TssE |
|  |  | - S | BCAM1082A BCAM1083 | putative exported phage protein putative transmembrane phage protein |
|  | None | - | BCAL0044 | putative transposase |
|  |  | - | BCAL0124 | flagellar regulon master regulator subunit FlhD |
|  |  | - | BCAL0434 | putative exported protein |
|  |  | - | **BCAL0594** | putative exported protein |
|  |  | - | BCAL1203 | conserved hypothetical protein (BcenGI5) |
|  |  | - | ***BCAL1535*** | putative membrane protein |
|  |  | - | BCAL3029 | putative alkane monooxygenase |
|  |  | - | BCAL3077 | putative membrane protein (BcenGI10) |
|  |  | - | **BCAM0164** | putative lipoprotein |
|  |  | - | BCAM0374 | conserved hypothetical protein |
|  |  | - | BCAM0520 | conserved hypothetical protein (pseudogene) |
|  |  | - | BCAM1053C | hypothetical phage protein (BcenGI12) |
|  |  | - | **BCAM1081** | hypothetical phage protein (BcenGI12) |
|  |  | - | BCAM1518 | conserved hypothetical protein |
|  |  | - | BCAM1890 | hypothetical phage protein (BcenGI13) |
|  |  | - | BCAM1892 | hypothetical phage protein (BcenGI13) |
|  |  | - | BCAM1893 | hypothetical phage protein (BcenGI13) |
|  |  | - | BCAM1901 | hypothetical phage protein (BcenGI13) |
|  |  | - | BCAM1904 | hypothetical phage protein (BcenGI13) |
|  |  | - | BCAM1918 | putative phage lysozyme (BcenGI13) |
|  |  | - | BCAM2126 | putative outer membrane porin |
|  |  | - | BCAM2169 | putative outer membrane autotransporter |
|  |  | - | **BCAM2274a** | conserved hypothetical protein |
|  |  | - | BCAM2348 | putative lipoprotein |
|  |  | - | ***BCAM2540*** | fenitrothion hydrolase protein FedB |
|  |  | - | BCAM2609 | putative exported protein |
|  |  | - | BCAM2654 | putative acetyltransferase |
|  |  | - | BCAM2764 | putative exported lipase |
|  |  | - | **BCAS0094** | putative membrane protein |
|  |  | - | **BCAS0633** | hypothetical protein |
|  |  | - | BCAS0661B | conserved hypothetical protein |
|  |  | - | pBCA080 | hypothetical protein |
|  |  | - - | ***BCAL1526 BCAL1527*** | putative flp type pilus assembly protein flp type pilus assembly protein |
|  |  | - - | BCAM1910 BCAM1911 | hypothetical phage protein (BcenGI13) hypothetical phage protein (BcenGI13) |
|  |  | - - | BCAS0684 BCAS0686 | conserved hypothetical protein (fragment) conserved hypothetical protein |
|  |  | - R | BCAM1569 BCAM1570 | putative BNR/Asp-box protein alcohol dehydrogenase |
|  |  | - S | BCAM1082A BCAM1083 | putative exported phage protein putative transmembrane phage protein |
|  |  | T - | BCAL1599b BCAL1601 | hypothetical phage protein (BcenGI7) hypothetical phage protein (BcenGI7) |
|  |  | E - | BCAM2486 BCAM2487 | putative membrane protein putative membrane protein |
|  |  | K - | BCAL1403 BCAL1403A | LysR family regulatory protein putative membrane protein |
|  |  | P - | BCAS0249 BCAS0250 | chromate resistance transport protein conserved hypothetical protein |

**^a^ Genes** were significantly more highly expressed in intracellular bacteria than in non-macrophage-exposed (NME) bacteria (log2 > 2, *p* < 0.05)

***Genes*** are in a transcriptional unit with genes significantly more highly expressed in intracellular bacteria than in NME bacteria (log2 > 2, *p* < 0.05)

Genes are more highly expressed by intracellular bacteria than in NME bacteria
