# Supplementary material for: Global changes in gene expression by the opportunistic pathogen Burkholderia cenocepacia in response to internalization by murine macrophages
Source: BMC Genomics. 2012 Feb 9;13:63. doi: 10.1186/1471-2164-13-63 (PMC3296584; doi:10.1186/1471-2164-13-63)
Supplement: Additional file 3 — Table S2-Genes with significantly higher or lower expression by intracellular B. cenocepacia. Genes included show greater than 2-fold change in expression (p < 0.05) in intracellular bacteria relative to non-macrophage-exposed bacteria. The first sheet contains genes with increased intracellular expression, the second genes with decreased intracellular expression. [file 1471-2164-13-63-S3.DOC]

**Table S2: Genes strongly repressed by intracellular *B. cenocepacia***

| **Classification** | **COG** | **Gene** | **Closest Gene** | **Known or putative function** | **p-value** | **Fold Change** |
| --- | --- | --- | --- | --- | --- | --- |
| Metabolism | Amino Acid Transport & Metabolism | BCAL2244 |  | urocanate hydratase (hutU) | 1.90E-02 | 28.10 |
|  |  | BCAL3197 |  | serine hydroxymethyltransferase (glyA) | 7.81E-03 | 39.82 |
|  |  | BCAM0746 |  | arginosuccinate synthase argG | 7.33E-03 | 11.03 |
|  |  | IG3_26003 | BCAS0024 | GntR family regulatory protein | 2.56E-02 | 17.55 |
|  |  | IG1_3436208 | BCAL3157 | putative ornithine cyclodeaminase | 1.16E-02 | 15.25 |
|  | Energy Production & Conversion | BCAL1214 |  | branched-chain alpha-keto acid dehydrogenase subunit E2 (bkdB) | 3.40E-02 | 15.59 |
|  |  | BCAL2143 |  | ubiquinol oxidase polypeptide I (cyoB) | 4.27E-03 | 80.60 |
|  |  | BCAM0042 |  | putative aldo/keto reductase | 3.61E-02 | 15.01 |
|  |  | BCAM2675 |  | putative cytochrome oxidase subunit II | 2.54E-02 | 31.91 |
|  | Carbohydrate Transport & Metabolism | BCAM2135 |  | major facilitator superfamily protein | 2.25E-02 | 11.71 |
|  | Coenzyme Transport & Metabolism | BCAL0264 |  | delta-aminolevulinic acid dehydratase (hemB) | 3.94E-03 | 29.36 |
|  |  | IG2_3145916 | BCAM2784 | aminotransferase | 1.03E-03 | 41.88 |
|  | Ion Transport & Metabolism | BCAL0740 |  | hypothetical protein | 4.60E-03 | 13.60 |
|  |  | BCAL1700 |  | ornibactin receptor (orbA) | 3.29E-02 | 10.01 |
|  |  | BCAL2299 |  | putative permease | 2.30E-02 | 29.76 |
|  |  | BCAL3297 |  | putative ferritin DPS-family DNA binding protein | 2.40E-02 | 25.61 |
|  |  | BCAM0827 |  | putative voltage gated chloride channel membrane protein | 3.43E-02 | 37.03 |
|  | Nucleotide Transport & Metabolism | BCAL2387 |  | uracil phosphoribosyltransferase (upp) | 6.46E-03 | 27.83 |
|  | Lipid Transport & Metabolism | BCAL1473 |  | succinyl-CoA:3-ketoacid-coenzyme A transferase subunit B (scoB) | 8.15E-04 | 27.84 |
|  |  | BCAM2430 |  | putative biotin carboxylase | 1.55E-02 | 18.92 |
|  | Biosynthesis & Catabolism | BCAL3184 |  | homogentisate 1,2-dixoygenase (hmgA) | 1.82E-02 | 65.59 |
|  |  | BCAM0906 |  | putative dienelactone hydrolase family protein | 3.14E-02 | 18.93 |
|  |  | BCAM2707 |  | putative FAA-hydrolase family protein | 3.21E-02 | 10.79 |

| Cellular Processes & Signalling | Signal Transduction Mechanisms | IG1_1435033 | BCAL1318 | putative nitrate regulatory protein | 6.16E-03 | 10.06 |
| --- | --- | --- | --- | --- | --- | --- |
|  |  | IG2_1573637 | BCAM1422 | putative cyclic nucleotide binding protein | 1.82E-02 | 10.46 |
|  | Trafficking, Secretion & Transport | BCAL2292 |  | putative biopolymer transport protein | 3.20E-02 | 13.59 |
|  | Posttranslational Modification, Chaperones | BCAL0468 |  | M48 family peptidase | 7.53E-03 | 20.30 |
|  |  | BCAL1995 |  | ATP-dependent protease ATP-binding subunit ClpX | 4.87E-02 | 10.68 |
|  |  | BCAL3192 |  | putative oxidoreductase | 3.75E-02 | 10.44 |
|  | Membrane Biogenesis | BCAL3135 |  | dTDP-D-glucose 4,6-dehydratase (rmiB) | 2.15E-02 | 16.63 |
|  |  | IG3_642613 | BCAS0591 | efflux system transport protein | 4.70E-02 | 10.87 |
| Information Storage & Processing | Transcription | BCAL1440 |  | LysR family regulatory protein | 5.77E-03 | 77.50 |
|  |  | BCAL1901 |  | transcription termination factor Rho | 1.80E-02 | 35.58 |
|  |  | BCAL2048 |  | GntR family regulatory protein | 4.11E-02 | 18.16 |
|  |  | BCAL2985 |  | GntR family regulatory protein | 9.85E-03 | 17.08 |
|  |  | BCAM0240 |  | N-acylhomoserine lactone dependent regulatory protein (cciR) | 1.37E-02 | 46.13 |
|  |  | BCAM1928 |  | putative transcription elongation factor | 1.23E-02 | 69.67 |
|  |  | BCAM1943 |  | MarR family regulatory protein | 2.54E-02 | 19.08 |
|  |  | IG3_26003 | BCAS0024 | GntR family regulatory protein | 2.56E-02 | 17.55 |
|  | Ribosomal Structure & Biogenesis | BCAL0484 |  | aspartyl/glutamyl-tRNA amidotransferase subunit A (gatA) | 3.38E-02 | 10.93 |
|  |  | IG1_1598102 | BCAL1448 | valyl-tRNA synthetase (valS) | 2.14E-02 | 11.04 |
| Poorly Characterized | General Function Prediction only | BCAL1414 |  | putative hydrolase | 1.45E-03 | 57.89 |
|  |  | BCAL1965 |  | putative lipoprotein | 1.77E-02 | 24.22 |
|  |  | BCAL2459 |  | putative O-methyltransferase | 1.25E-02 | 16.73 |
|  |  | BCAL2816 |  | S-formylglutathione hydrolase | 4.86E-02 | 25.61 |
|  |  | BCAL2851 |  | putative hydrolase | 3.98E-02 | 44.20 |
|  |  | BCAM0165 |  | hypothetical protein | 3.52E-02 | 15.56 |
|  |  | BCAS0703 |  | putative short chain dehydrogenase | 1.96E-02 | 31.93 |
|  | Function Unknown | BCAL1177 |  | putative fusaric acid resistance transporter protein | 2.96E-02 | 18.42 |
|  |  | BCAL1606 |  | hypothetical protein | 1.90E-02 | 10.88 |
|  |  | BCAM0770 |  | hypothetical protein | 2.69E-03 | 50.40 |
|  |  | BCAM1233 |  | hypothetical protein | 1.07E-02 | 11.73 |
|  |  | BCAM2700 |  | hypothetical protein | 6.22E-04 | 19.09 |
|  |  | BCAS0667 |  | hypothetical protein | 4.98E-02 | 11.40 |
|  |  | IG2_1044007 | BCAM0492 | putative transmembrane peptidase | 1.34E-02 | 27.15 |
|  |  | IG2_2036821 | BCAM1818 | hypothetical protein | 3.53E-02 | 21.39 |
|  |  | pBCA008 |  | hypothetical protein | 4.52E-03 | 35.68 |
|  | None | BCAL0810 |  | putative PTS system, EIIA 2 component (pseudogene) | 2.78E-03 | 11.22 |
|  |  | BCAL1316 |  | hypothetical protein | 1.73E-02 | 10.64 |
|  |  | BCAL1418 |  | major facilitator superfamily protein | 2.33E-02 | 19.25 |
|  |  | BCAL1875 |  | hypothetical protein | 2.24E-02 | 11.90 |
|  |  | BCAL2025 |  | hypothetical protein | 1.91E-02 | 37.61 |
|  |  | BCAL2300 |  | hypothetical protein | 1.22E-02 | 13.82 |
|  |  | BCAL2461 |  | hypothetical protein | 4.14E-02 | 10.33 |
|  |  | BCAL2998 |  | transglycosylase associated protein | 4.66E-03 | 14.72 |
|  |  | BCAM0329 |  | hypothetical protein | 9.26E-03 | 52.46 |
|  |  | BCAM0330 |  | putative lipoprotein | 2.10E-02 | 22.02 |
|  |  | BCAM1030 |  | hypothetical protein | 1.90E-02 | 12.71 |
|  |  | BCAM2623 |  | hypothetical protein | 2.44E-02 | 14.17 |
|  |  | BCAM2685 |  | hypothetical protein | 1.70E-03 | 59.50 |
|  |  | BCAS0414 |  | hypothetical protein | 2.68E-02 | 20.91 |
|  |  | IG1_2896142 | BCAL2633 | putative DNA-binding protein | 1.01E-03 | 10.43 |
|  |  | IG1_300118 | BCAL0272 | putative lipoprotein | 1.98E-02 | 11.50 |
|  |  | IG1_803639 | BCAL0740 | hypothetical protein | 5.37E-03 | 28.34 |
|  |  | IG2_2129074 | BCAM1916 | transposase | 2.64E-02 | 13.36 |
|  |  | pBCA029 |  | hypothetical protein | 3.19E-03 | 14.73 |
|  |  | pBCA045 |  | hypothetical protein | 3.08E-02 | 15.11 |
|  |  | pBCA046 |  | putative traE conjugative transfer protein | 1.76E-02 | 25.95 |
|  |  | pBCA067 |  | hypothetical protein | 2.75E-02 | 22.44 |
|  |  | pBCA077 |  | hypothetical protein | 1.03E-02 | 90.16 |
|  |  | BCALr1899 |  | bacterial signal recognition particle RNA | 2.05E-02 | 11.02 |

| Cellular Processes & Signalling | Signal Transduction Mechanisms | BCAL2913 |  | sigma-54 interacting regulatory protein | 2.37E-02 | 31.92 |
| --- | --- | --- | --- | --- | --- | --- |
|  |  | BCAM0319 |  | putative universal stress protein | 4.04E-02 | 14.07 |
|  |  | BCAM1554 |  | putative diguanylate cyclase | 1.45E-02 | 14.79 |
|  |  | BCAM2837 |  | two-component regulatory system, response regulator | 2.30E-02 | 210.68 |
|  |  | BCAL0134 |  | chemotaxis-specific methylesterase cheB1 | 8.99E-03 | 17.39 |
|  | Trafficking, Secretion & Transport | IG1_1836014 | BCAL1678 | putative outer membrane usher protein precursor | 1.70E-02 | 41.61 |
|  |  | BCAM0561 |  | transport protein | 3.57E-03 | 73.84 |
|  | Posttranslational Modification, Chaperones | BCAS0302 |  | conserved hypothetical protein | 4.95E-02 | 11.69 |
|  | Membrane Biogenesis | BCAM0104 |  | putative dihydrodipicolinate synthase | 1.51E-02 | 19.41 |
|  |  | BCAL1043 |  | glucarate dehydratase gudD | 3.64E-02 | 14.23 |
|  | Cell Motility | BCAL0113 |  | B-type flagellar hook-associated protein 2 fliD1 | 3.96E-02 | 11.59 |
|  |  | BCAL0568 |  | flagellar basal-body rod protein FlgF | 3.09E-02 | 35.69 |
|  |  | BCAL0134 |  | chemotaxis-specific methylesterase cheB1 | 8.99E-03 | 17.39 |
|  |  | IG1_1836014 | BCAL1678 | putative outer membrane usher protein precursor | 1.70E-02 | 41.61 |
|  | Defense Mechanisms | BCAM2141 |  | ABC transporter ATP-binding membrane protein | 2.42E-02 | 37.63 |
| Information Storage & Processing | Transcription | BCAL2561 |  | LysR family regulatory protein | 9.30E-03 | 10.55 |
|  |  | BCAL2693 |  | LysR family regulatory protein | 4.99E-02 | 27.77 |
|  |  | BCAL3006 |  | cold shock-like protein cspA | 1.09E-02 | 12.83 |
|  |  | BCAM0417 |  | LuxR superfamily regulatory protein | 1.30E-02 | 13.08 |
|  |  | BCAL3335 |  | DNA-binding protein Fis | 3.63E-02 | 12.84 |
|  | Replication, Recombination & Repair | BCAL3335 |  | DNA-binding protein Fis | 3.63E-02 | 12.84 |

| Poorly Characterized | General Function Prediction only | BCAL1043 |  | glucarate dehydratase gudD | 3.64E-02 | 14.23 |
| --- | --- | --- | --- | --- | --- | --- |
|  |  | BCAL2689 |  | putative short-chain type dehydrogenase/reductase | 2.91E-02 | 11.84 |
|  |  | BCAL1112 |  | putative phosphodiesterase | 4.90E-02 | 10.87 |
|  |  | BCAM0949 |  | lipase | 1.89E-02 | 12.84 |
|  |  | BCAM1244 |  | putative phosphonopyruvate decarboxylase | 6.19E-03 | 14.19 |
|  |  | BCAS0627 |  | putative lipoprotein | 1.69E-02 | 10.80 |
|  |  | IG2_429106 | BCAM0382 | putative lipoprotein | 1.06E-02 | 11.66 |
|  | Function Unknown | BCAL1956 |  | putative lipoprotein | 4.55E-02 | 11.44 |
|  |  | BCAM0816 |  | conserved hypothetical protein | 2.71E-02 | 11.56 |
|  | None | BCAL0193 |  | putative exported protein | 1.33E-02 | 14.01 |
|  |  | BCAL0352 |  | metallo peptidase, subfamily M15C | 4.93E-02 | 14.56 |
|  |  | BCAL0932 |  | conserved hypothetical protein | 3.84E-02 | 16.90 |
|  |  | BCAL1577 |  | hypothetical phage protein | 4.41E-02 | 18.63 |
|  |  | BCAM0314 |  | conserved hypothetical protein | 1.04E-03 | 163.22 |
|  |  | BCAM0468 |  | conserved hypothetical protein | 6.23E-03 | 19.90 |
|  |  | BCAM2023 |  | conserved hypothetical protein | 4.39E-02 | 11.56 |
|  |  | BCAM2542 |  | fenitrothion hydrolase protein FedA | 3.13E-02 | 19.10 |
|  |  | BCAM2740 |  | conserved hypothetical protein | 4.14E-02 | 34.59 |
|  |  | BCAS0094 |  | putative membrane protein | 1.35E-02 | 20.16 |
|  |  | BCAS0681 |  | putative transposase (pseudogene) | 2.54E-02 | 13.57 |
|  |  | IG2_1793182 | BCAM1611 | putative exported protein | 9.90E-03 | 31.41 |
|  |  | IG2_369070 | BCAM0314 | conserved hypothetical protein | 5.60E-03 | 51.99 |
|  |  | IG3_607288 | BCAS0550 | hypothetical phage protein - BcepMu5 | 4.55E-02 | 22.11 |
