# Supplementary material for: Global changes in gene expression by the opportunistic pathogen Burkholderia cenocepacia in response to internalization by murine macrophages
Source: BMC Genomics. 2012 Feb 9;13:63. doi: 10.1186/1471-2164-13-63 (PMC3296584; doi:10.1186/1471-2164-13-63)
Supplement: Additional file 4 — Table S3-In vivo essential genes with increased expression in intracellular bacteria. [file 1471-2164-13-63-S4.DOC]

**Table S3 – *In vivo* essential genes with increased expression in intracellular bacteria**

| Tag^a^ | Gene designation^b^ | Known or putative function |
| --- | --- | --- |
| Selectively captured sequences | | |
| 7C6 | BCAL0770 | putative DNA polymerase III alpha subunit |
| 37G5 | BCAL0345^c^ | conserved hypothetical protein |
| 34C6 | BCAL0352^c^ | metallo peptidase, subfamily M15C |
| 34A1 | BCAL1536 | putative sigma-54 related transcriptional regulatory protein |
| 36B4 | BCAL3240 | putative capsular polysaccharide transporter ATP-binding protein |
| 31B3 | BCAM0992 | putative DNA methylase |
| 31C5 | BCAS0258 | GntR family regulatory protein |
|  |  |  |
| Microarray | | |
| 4G5 | BCAL0154 | histone-like nucleoid-structuring (H-NS) protein |
| 34C4 | BCAL0339 | putative type VI secretion system protein TssJ |
| 1A5 | BCAL0347 | protease associated ATPase ClpB |
| 34C6 | **BCAL0352** | metallo peptidase, subfamily M15C |
| 15B2 | BCAL0423 | chromosomal replication initiation protein |
| 38H2 | BCAL1122 | conserved hypothetical protein |
| 1H4 | BCAL1318 | putative nitrate regulatory protein |
| 9C1 | BCAL1850 | putative dehydrogenase |
| 38C6  39H4 | BCAL2732 | cold shock-like protein |
| 32D2 | BCAL3122 | glycosyltransferase |
| 38C2 | **BCAL3125** | glycosyltransferase |
| 3A6 | BCAL3173 | xanthine dehydrogenase |
| 4H4 | BCAL3178 | LysR family regulatory protein |
| 10F1  33F9 | **BCAL3228** | hypothetical protein |
| 40H2 | BCAL3258 | tetracycline repressor protein |
| 20D2 | BCAL3287 | putative FAD-binding oxidase |
| 33G2 | BCAM0020 | putative membrane protein |
| 33B1 | BCAM0179 | putative mechanosensitive ion channel protein |
| 28D8 | **BCAM1011** | putative acetyltransferase |
| 33G4 | BCAM1478 | aromatic amino acid aminotransferase |
| 28D5 | BCAM1679 | putative lysylphosphatidylglycerol synthetase |
| 40C1 | BCAM1861 | calcineurin-like phosphoesterase |
| 6E3 | BCAM1867 | putative Mg(2+) transport ATPase |
| 18D2 | **BCAM2150** | transcriptional activator FtrA |
| 31C5 | BCAS0257 | putative acetyltransferase |
| 31G1 | BCAS0695 | putative phospholipid-binding exported protein |

^a^Tag assigned to mutant in STM *in vivo* study [38]

^b^Genes in bold were significantly more highly expressed in intracellular bacteria than in non-macrophage-exposed bacteria (log2 > 2, p<0.05)

^c^Immediately adjacent gene identified by SCOTS
