# Supplementary material for: Global changes in gene expression by the opportunistic pathogen Burkholderia cenocepacia in response to internalization by murine macrophages
Source: BMC Genomics. 2012 Feb 9;13:63. doi: 10.1186/1471-2164-13-63 (PMC3296584; doi:10.1186/1471-2164-13-63)
Supplement: Additional file 5 — Figure S2-Fold change gene expression of selected SCOTS-identified genes measured by qRT-PCR. [file 1471-2164-13-63-S5.DOC]

**
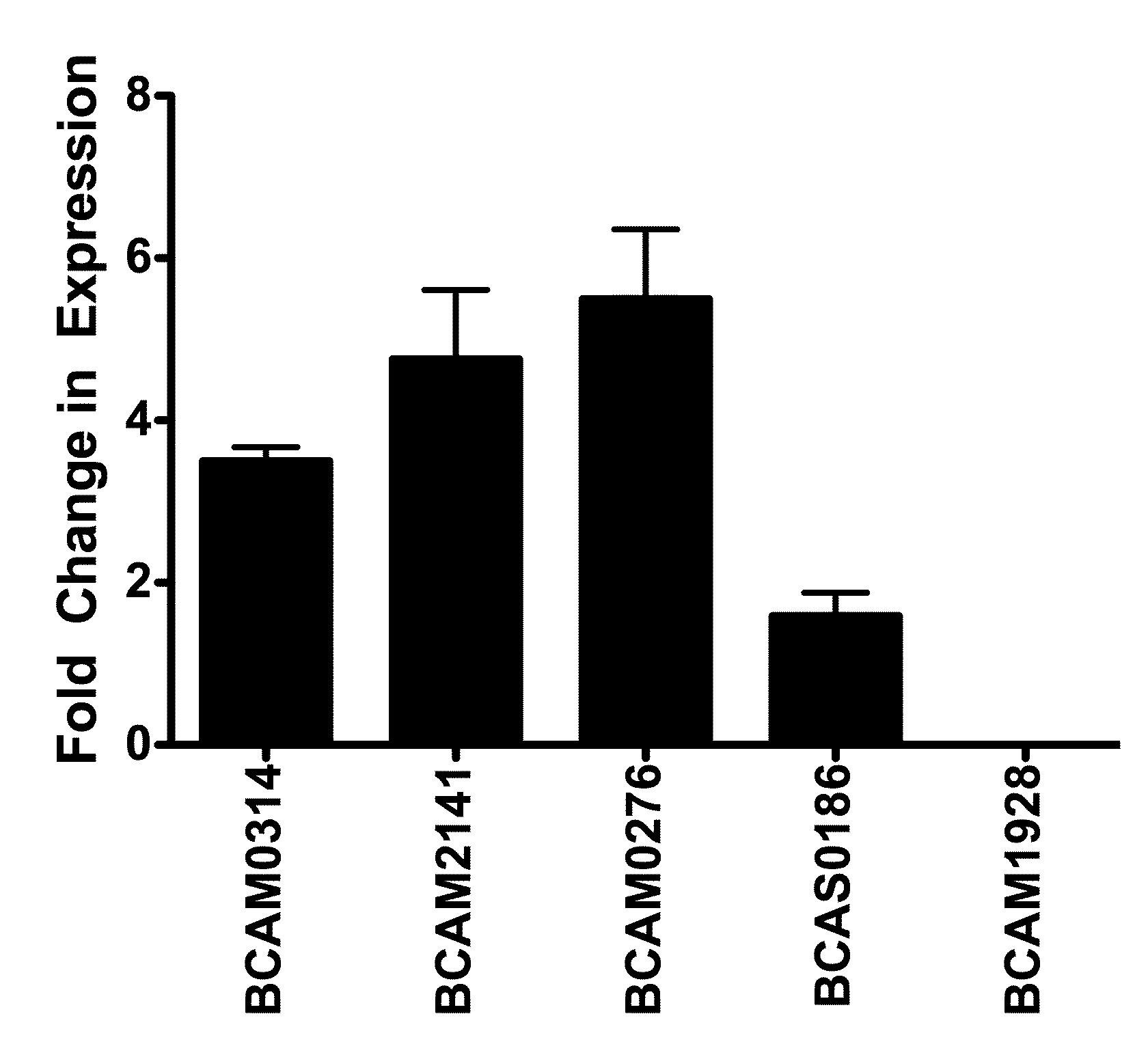
**

**Figure S2 – Fold change gene expression of selected SCOTS-identified genes measured by qRT-PCR.** Fold change represents the ratio of expression by intracellular bacteria to expression by non-macrophage-exposed bacteria, each relative to expression of the reference gene *rpoD*.
