# Supplementary material for: Global changes in gene expression by the opportunistic pathogen Burkholderia cenocepacia in response to internalization by murine macrophages
Source: BMC Genomics. 2012 Feb 9;13:63. doi: 10.1186/1471-2164-13-63 (PMC3296584; doi:10.1186/1471-2164-13-63)
Supplement: Additional file 6 — Figure S3-ΔBCAL0124 and ΔBCAM2837 are defective in motility. [file 1471-2164-13-63-S6.DOC]

**
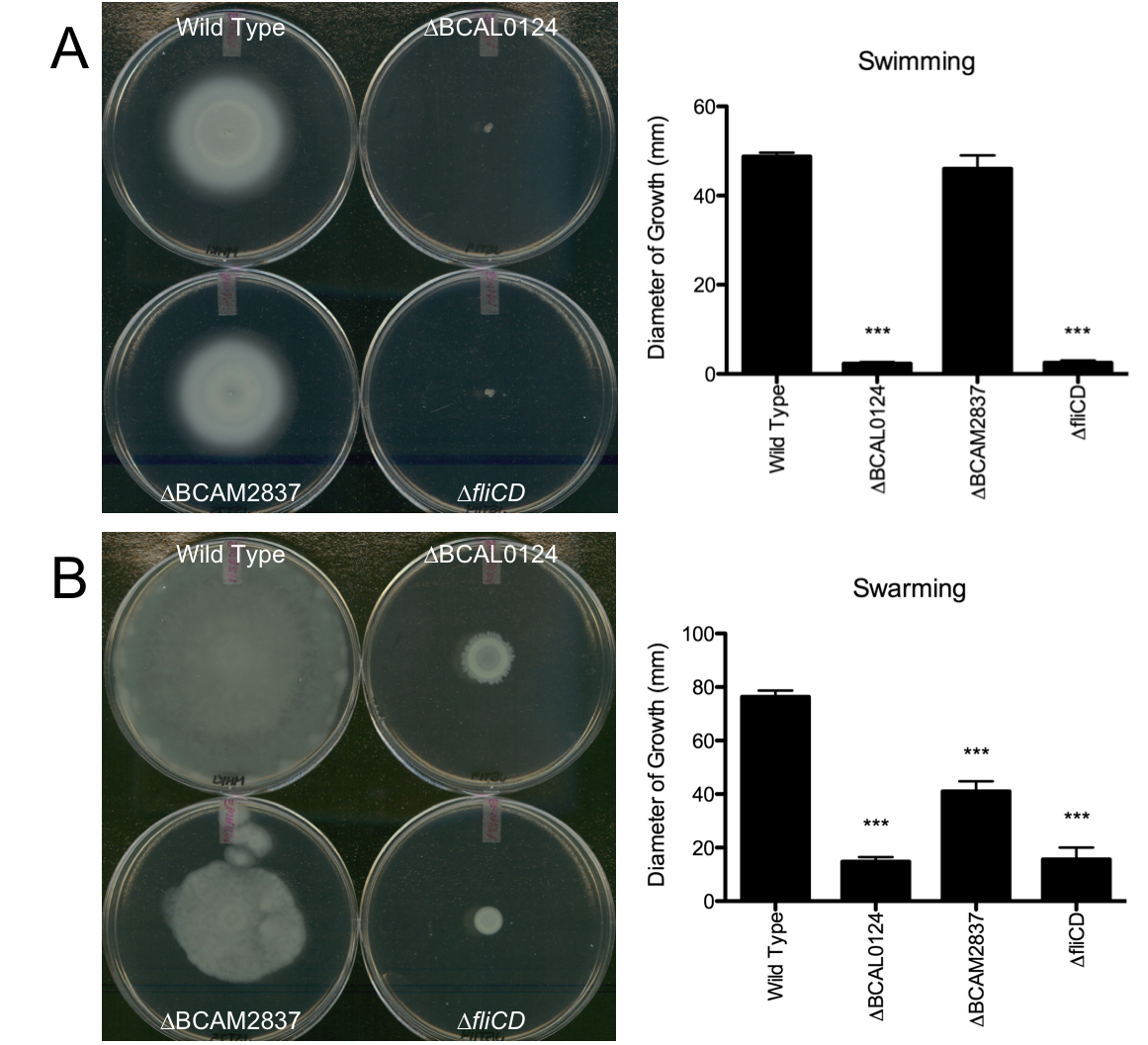
**

**Figure S3 – ∆BCAL0124 and ∆BCAM2837 are defective in motility.** Bacterial cultures were adjusted to an OD_600_ of 1.0; 2 μL was stab-inoculated into [LB 0.3% agar] to observe swimming motility (A), and 2 μL was drop-inoculated onto [Nutrient Broth 0.2% glucose 0.5% agar] to observe swarming motility (B). Error bars in quantitation represent the standard error of at least two independent experiments observed 24 hours post-inoculation. Significance was determined using one-way ANOVA and Dunnett’s Multiple Comparison Test. Mutants demonstrating significant difference from the wild-type are indicated (****p*<0.001).
