# Supplementary material for: Global changes in gene expression by the opportunistic pathogen Burkholderia cenocepacia in response to internalization by murine macrophages
Source: BMC Genomics. 2012 Feb 9;13:63. doi: 10.1186/1471-2164-13-63 (PMC3296584; doi:10.1186/1471-2164-13-63)
Supplement: Additional file 7 — Figure S4-Flagellin is necessary for efficient bacterial entry into macrophages. [file 1471-2164-13-63-S7.DOC]

**
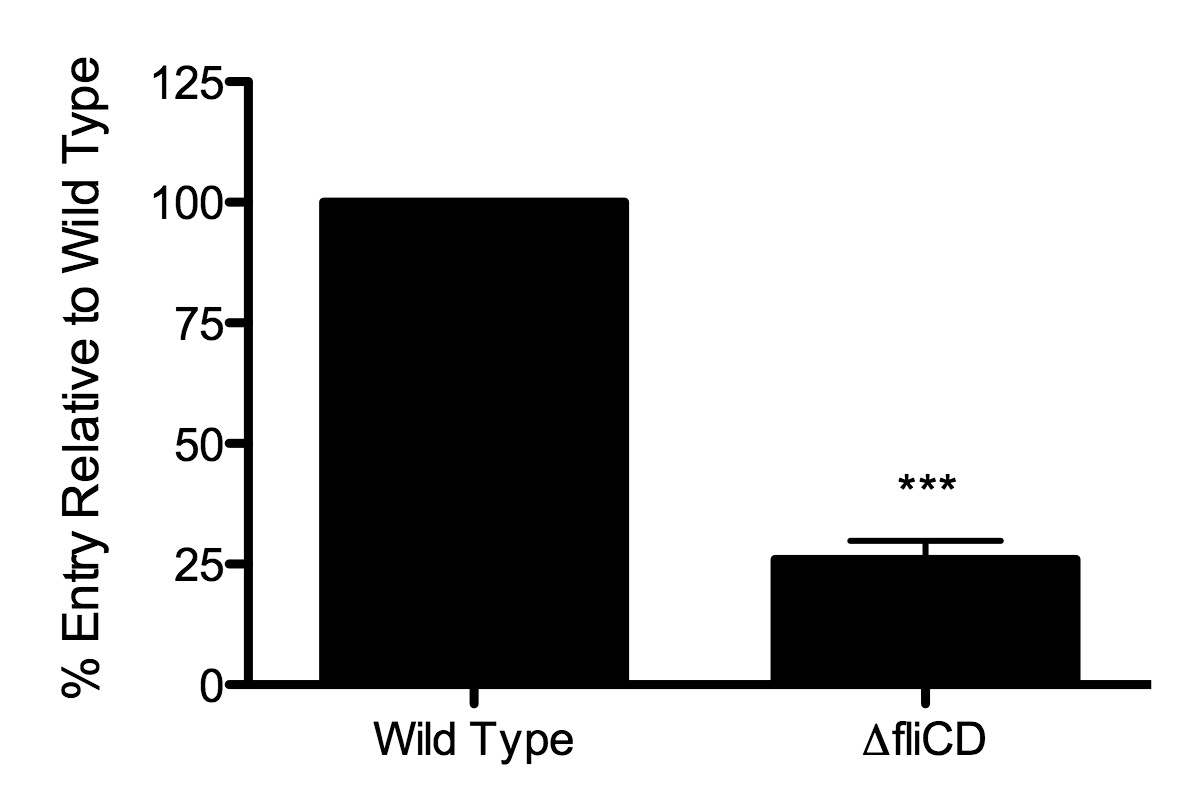
**

**Figure S4 – Flagellin is necessary for efficient bacterial entry into macrophages.** Entry was calculated relative to initial inoculum, and normalized relative to the parental control, set at 100% entry. Error bars indicate the standard error. Significance was determined using Student’s t-test. ∆*fliCD* is significantly different from the parental strain with *p* < 0.001.
