# Supplementary material for: Global changes in gene expression by the opportunistic pathogen Burkholderia cenocepacia in response to internalization by murine macrophages
Source: BMC Genomics. 2012 Feb 9;13:63. doi: 10.1186/1471-2164-13-63 (PMC3296584; doi:10.1186/1471-2164-13-63)
Supplement: Additional file 8 — Table S4-Oligonucleotide primers used in this study. [file 1471-2164-13-63-S8.DOC]

**Table S4 – Oligonucleotide primers used in this study**

| Primer name | 5’-3’ primer sequence^a^ | Restriction enzyme^b^ |
| --- | --- | --- |
| SCOTS primers | | |
| I-3025 | TGCTCTAGACGTCCTGAATTCCGTANNNNNNNNN | XbaI |
| N-3017 | GATGTGTCGACTCAGGATCCATAGGNNNNNNNNN | BamHI |
| I-3032 | TGCTCTAGACGTCCTGAATTCCGTA | XbaI |
| N-3033 | GATGTGTCGACTCAGGATCCATAGG | BamHI |
| M13F | GTAAAACGACGGCCAGT | N/A |
| M13R | CAGGAAACAGCTATGAC | N/A |
|  |  |  |
| qPCR primers | | |
| BCAM0314-F | GCATCATGGGATTTATGTCG | N/A |
| BCAM0314-R | GGCCTCTGTTCCGTCGTA | N/A |
| BCAM2141-F | GAGCAGGTCGGATACAGCTT | N/A |
| BCAM2141-R | GCGTGTCGTTGAACAGGAT | N/A |
| BCAM0276-F | AGCATCATGGTCGCAGTAGA | N/A |
| BCAM0276-R | GCAGGATCGTCTTGCCATA | N/A |
| BCAS0186-F | TCTCGCAGAAGGATGTGTTG | N/A |
| BCAS0186-R | AGCCACGTTTCCATGTAAGG | N/A |
| BCAM1928-F | GTCACGATGAACTCGCAGAT | N/A |
| BCAM1928-R | GCTGCATCACCTTGACCAT | N/A |
| rpoD-F | GAGATGAGCACCGATCACAC | N/A |
| rpoD-R | CCTTCGAGGAACGACTTCAG | N/A |
|  |  |  |
| Deletion primers | | |
| BCAL0124-UF | TCCTCTAGAGGTTTACAGACAAATCCTGC | XbaI |
| BCAL0124-UR | TTTAATCGATAGGTCGAAGGCGTCCGCTG | ClaI |
| BCAL0124-DR | TTTTGAATTCCTTGTACAGCTTGATCAGAC | EcoRI |
| BCAL0124-DF | TTTTATCGATCACTGAGCATTTCGCTGGTA | ClaI |
| BCAS0186-UF | TTTTTCTAGAGTGCTCTATATCGAAGGCTC | XbaI |
| BCAS0186-UR | TTTAATCGATAGGCATCGGCAGATCCCA | ClaI |
| BCAS0186-DF | TTTTATCGATCTTACATGGAAACGTGGCTC | ClaI |
| BCAS0186-DR | TTTTGAATTCGGCGACGTCAGAACGTG | EcoRI |
| BCAM2837-UF | TTTTTCTAGACTTTGCCGATTGTAATTGCC | XbaI |
| BCAM2837-UR | TTTTATCGATTGTTTCCAGGACCTGATATC | ClaI |
| BCAM2837-DF | TTTTATCGATTCGATCCTCGACGAACTC | ClaI |
| BCAM2837-DR | TTTAGAATTCATCAGCCGGATCGAATCCT | EcoRI |
| BCAM1679-UF | TTTATCTAGACTGAACACGTTATGTCTTCC | XbaI |
| BCAM1679-UR | TATTATCGATGACGAGCGCGAGATAGCTGA | ClaI |
| BCAM1679-DF | TATAATCGATACGCAGTCGGTGATGCTGGT | ClaI |
| BCAM1679-DR | TATAGAATTCGAAGCGCCAGACCATCAGG | EcoRI |
| BCAL1726-UF | TTTTTCTAGATATAGTCAAGCATCGGTGGT | XbaI |
| BCAL1726-UR | TATAATCGATACACGAAACACGACGCACT | ClaI |
| BCAL1726-DF | CGCACGATTGGTCGCATCGAT | ClaI^c^ |
| BCAL1726-DR | TATAGAATTCCGATTCGATTCCTGCGT | EcoRI |
| BCAM0276-UF | TAATTCTAGATGGCACACGACCGAACAC | XbaI |
| BCAM0276-UR | TATTATCGATTCTACTGCGACCATGATGCT | ClaI |
| BCAM0276-DF | TATTATCGATTGATCGGCACACACGGA | ClaI |
| BCAM0276-DR | TATAGAATTCCCATTGACTGCGGACGAA | EcoRI |
| BCAM0411-UF | TATTTCTAGATCATCGAGTGGAACCGCA | XbaI |
| BCAM0411-UR | TTTTATCGATGACACGACCATCACGAG | ClaI |
| BCAM0411-DF | TTTTATCGATTTCATCATCGAGCAGGAC | ClaI |
| BCAM0411-DR | TTTAGAATTCAATGTCCTCGGAAATCTTC | EcoRI |
| BCAM2446-UF | TTTTTCTAGATCAGAACTTCTGGCGCAG | XbaI |
| BCAM2446-UR | TTTTATCGATCAGTATCACAACGTCACGC | ClaI |
| BCAM2446-DF | TTTAATCGATAGATCCTCGGAACCCTTGA | ClaI |
| BCAM2446-DR | TTCAGAATTCACATACAACATGAACAAGCA | EcoRI |
| BCAM0434-5-UF | TTTTTCTAGATGACAAGGCAATCACTGATG | XbaI |
| BCAM0434-5-UR | TTTTATCGATTTTGTCGAAGTGCTGGTT | ClaI |
| BCAM0434-5-DF | TTTTATCGATGAAGTCATGCAGCGCAT | ClaI |
| BCAM0434-5-DR | TTTTGAATTCTCATCTTCATCGTGATTCTC | EcoRI |
|  |  |  |
| Complementation Primers | | |
| BCAL0124-cF | TTTTTTCATATGCCAAAGCAAAGCTCTTTAAC | NdeI |
| BCAL0124-cR | TTTTTCTAGAGACAGACGCAAGGCGGGA | XbaI |
| BCAL0340-cF | TTTTTTCATATGTCTCAACGATTTAAAACTCA | NdeI |
| BCAL0340-cR | TTTTTCTAGAGTGGTTATCGCGTCAGTTGC | XbaI |
| BCAL1726-cF | TTTTCATATGCCCGATTCGATTCCTGCG | NdeI |
| BCAL1726-cR | TTTTTCTAGAGTAGATTTCCTGCCCGTC | XbaI |
| BCAM0411-cF | TTTTTTCATATGGAGTGGAACCGCAGGACAA | NdeI |
| BCAM0411-cR | TTAATCTAGAAATGTCCTCGGAAATGTTCG | XbaI |
|  |  |  |

^a^ Restriction endonuclease sites incorporated into the oligonucleotide sequences are underlined

^b^ N/A indicates the absence of a restriction site

^c^ native ClaI site in chromosome
